# ULBP2 CAR-T cells enhance gastric cancer immunotherapy by inhibiting CAF activation

**DOI:** 10.1038/s41419-025-07905-5

**Published:** 2025-08-08

**Authors:** Wentao Zhang, Wen Ren, Shuyan Guo, Haobo Han, Weiwen Cai, Haowen Bai, Long Li, Xiangyan Jiang, Xin Zheng, Tiansheng Zhang, Yan Wang, Huili Ye, Hongtai Cao, Wengui Shi, Huinian Zhou, Zeyuan Yu, Long Qin, Zuoyi Jiao

**Affiliations:** 1 Cuiying Biomedical Research Center, The Second Hospital & Clinical Medical School, Lanzhou University, Lanzhou, Gansu China; 2 Department of General Surgery, The Second Hospital & Clinical Medical School, Lanzhou University, Gansu, China; 3 Student Affairs Office, The Second Hospital & Clinical Medical School, Lanzhou University, Gansu, China; 4 Lanzhou Huazhitiancheng Biotechnologies Co., Ltd, Lanzhou, Gansu China; 5 Gansu Province High-Altitude High-Incidence Cancer Biobank, The Second Hospital & Clinical Medical School, Lanzhou University, Gansu, 730030 China

**Keywords:** Translational research, Immunosuppression

## Abstract

Gastric cancer (GC) is characterised by a dense stromal microenvironment, lack of therapeutic targets, and limited effective treatment options, collectively leading to a poor prognosis. Here, we identify UL16 binding protein 2 (ULBP2) as a potential therapeutic target in GC. Mechanistically, ULBP2 overexpression activates the TGF-β signalling pathway, promoting the activation of cancer-associated fibroblasts (CAFs) and tumor progression in GC. Furthermore, we developed ULBP2 CAR-T cells and assessed their therapeutic potential in GC cell lines, organoids, cell line-derived xenograft (CDX) and patient-derived xenograft (PDX) mouse models. We showed that ULBP2 CAR-T cells effectively eliminated GC cell lines and organoids and, either alone or in combination with an anti-PD-1 antibody, significantly inhibited tumor growth and prolonged survival in both CDX and PDX mouse models. In conclusion, ULBP2 contributes to GC progression by promoting TGF-β mediated CAF activation, which collectively reinforce the dense stromal microenvironment. Targeting ULBP2 suppresses tumor growth, reduces stromal deposition, and promotes T cell infiltration, thereby enhancing the efficacy of immunotherapy in GC.

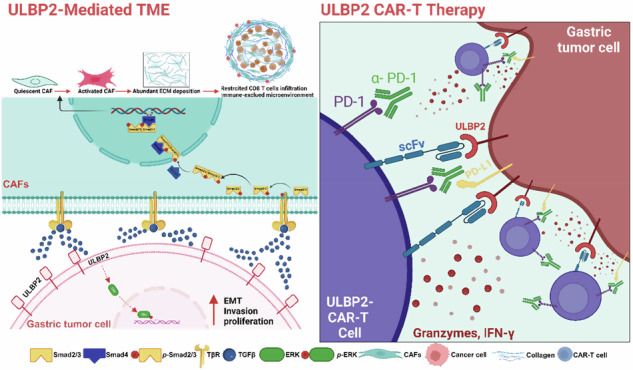

## Introduction

Gastric cancer (GC) ranks fifth globally in terms of tumor incidence and cancer-related deaths, with the highest incidence rates observed in Eastern Asia [1]. Owing to its delayed diagnosis, high metastatic potential, and dense stromal microenvironment, GC has a poor prognosis [2]. The extracellular matrix (ECM) is a complex acellular macromolecular network that drives cancer progression and immunotherapy resistance [3]. PD-1 inhibitors combined with chemotherapy are the preferred first-line therapy for GC, and pembrolizumab plus chemotherapy significantly improves overall survival (OS) from 11.5 to 12.9 months, compared with chemotherapy alone [4]. Although immunotherapy has revolutionised GC treatment, durable responses remain limited because of the dense stromal microenvironment, which facilitates immune evasion and contributes to resistance to both conventional and immune therapies [5].

Chimeric antigen receptor (CAR) T cells are approved for use in patients with B-cell malignancies and multiple myeloma; however, their efficacy against most solid tumors remains elusive [6–8]. Claudin 18.2 (CLDN18.2) is highly expressed in GC and is one of the most popular therapeutic targets [9]. The Phase 1 NCT03874897 trial found that CT041, a CLDN18.2-specific CAR-T cells, achieved an objective response rate (ORR) of 54.9% and a disease control rate (DCR) of 96.1% in 59 GC patients, with a median progression-free survival (mPFS) of 5.8 months (95% CI: 4.1, 8.0) and a median overall survival (mOS) of 9.0 months (95% CI: 7.0, 11.9), respectively; the OS rate at 12 months was 37.3% (95% CI: 25.2, 49.4) [10]. A number of studies have found that uPAR, HER2, Mesothelin, CD133, and Trop2/PD-L1 CAR-T cells show promising efficacy for the treatment of GC [11–15]. However, the slow progress of CAR-T therapy in GC underscores the urgent need to identify alternative targets that can modulate the dense stromal microenvironment to develop novel therapeutic strategies.

Natural killer group 2D (NKG2D) receptor is expressed in NK cells as well as certain T cells, such as NKT cells, CD8^+^ T cells, and γδT cells. NKG2D ligands (NKG2DL), which are specifically expressed in most tumor cells, bind to NKG2D receptors to activate NK cells and induce tumor cell destruction. However, various mechanisms inhibit the activation of the NKG2D receptor, NKG2DL, to enable immune escape of tumor cells [16, 17]. NKG2D CAR-T cells have satisfactory treatment efficacy and potential application value in several solid tumors, including glioblastoma, hepatocellular carcinoma, breast cancer, and GC [18–22]. However, although NKG2D is a highly conserved receptor, NKG2DLs are diverse, including MICA, MICB, and UL16 binding proteins (ULBPs), leading to suboptimal efficacy and thus necessitating further investigation. UL16 binding protein 2 (ULBP2), an MHC class I-related molecule, is minimally expressed in normal tissues but is highly upregulated in tumor tissues, including pancreatic and colorectal cancers [23, 24]. A recent study showed that ULBP2 expression correlates with the immunosuppressive TME and immunotherapy in breast cancer [25]. Therefore, it is essential to investigate the role of ULBP2 in the immunosuppressive microenvironment of GC and explore potential therapeutic strategies.

We, therefore, aimed to identify the oncogenic role that ULBP2 plays in GC as a potential therapeutic target. ULBP2 promotes the formation of a dense stromal microenvironment in GC by activating cancer-associated fibroblasts (CAFs) and driving collagen deposition through the TGF-β signalling pathway. Furthermore, we developed a novel ULBP2 CAR-T cell line that effectively killed ULBP2-expressing cells and patient-derived GC organoids in vitro. Treatment with ULBP2 CAR-T cells and anti-PD-1 showed remarkable antitumor efficacy, enhancing the survival of GC cell-line-derived xenografts (CDX) and GC patient-derived xenograft (PDX) mouse models. Our study offers new promising therapeutic strategies for the treatment of GC.

## Results

### ULBP2 is overexpressed in GC and correlates with collagen deposition

**Fig. 1:**
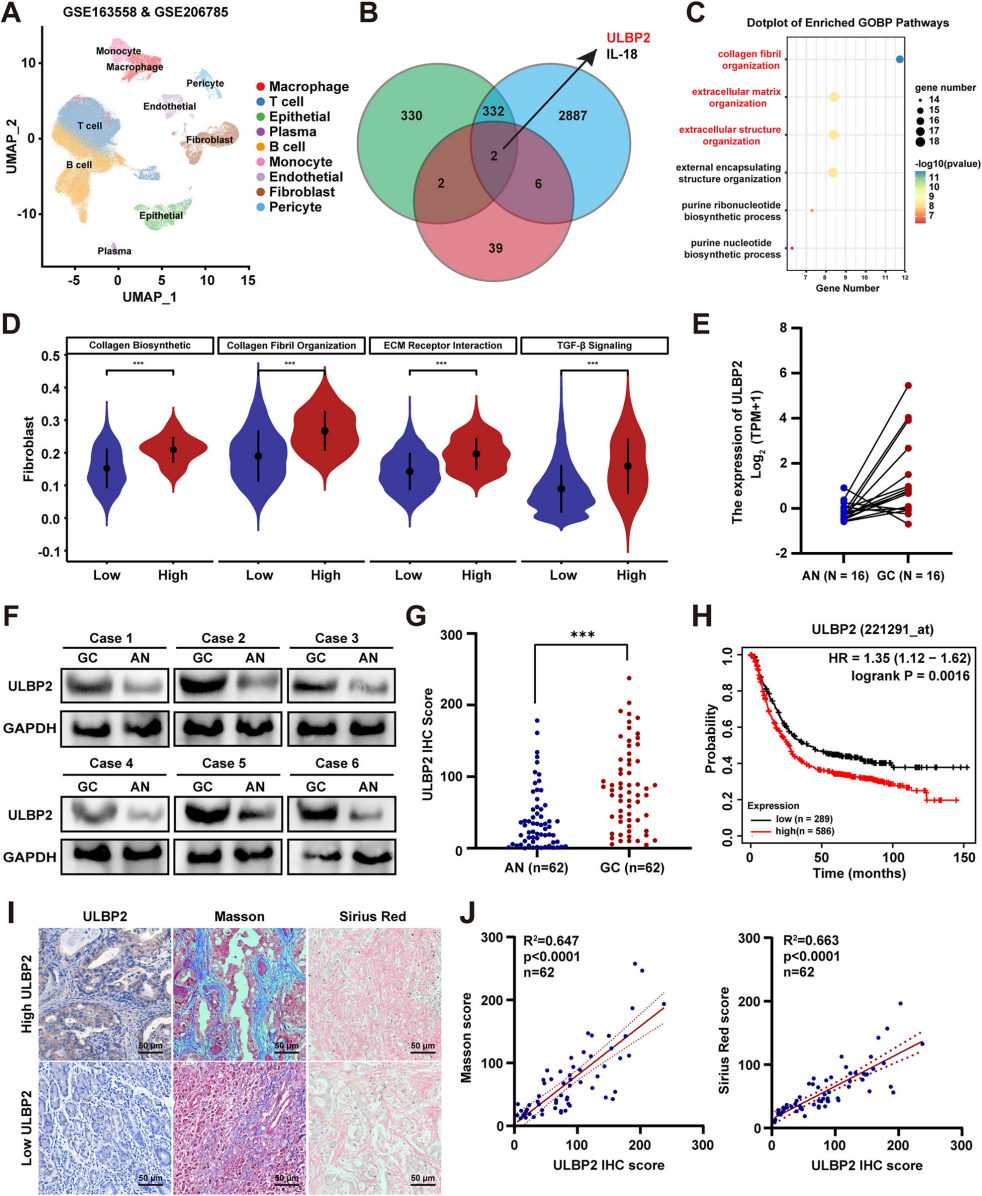
ULBP2 is overexpressed in GC and correlated with collagen deposition. **A** The t-Distributed stochastic neighbour embedding (t-SNE) plot showing clustering information from GSE163558 and GSE206785. **B** Venn diagram showing the overlap between mRNA microarray, GSE163558 and GSE206785, upregulated genes associated with ECM, and Immunology Database and Analysis Portal (ImmPort) database. **C** Gene Ontology (GO) analysis showing enriched pathway terms for genes in GSE163558 and GSE206785. **D** ULBP2 expression in fibroblasts revealed a correlation with collagen biosynthesis, collagen fibril organisation, ECM-receptor interaction, and TGF-β signalling. **E** ULBP2 mRNA expression of GC and corresponding adjacent normal tissues analyzed by mRNA microarrays (n = 16). **F** Immunoblotting of ULBP2 in the tumors and corresponding adjacent normal tissues from GC patients (n = 6). **G** Quantification of ULBP2 expression by immunohistochemistry (IHC) in human tissue microarrays (TMAs) from 62 gastric patients. **H** Kaplan–Meier survival analysis of overall survival (OS). **I** Representative images of gastric tissues with high ULBP2 expression compared to low-expression tissues, stained by IHC, Masson, and Sirius Red stains. **J** Scatter plots of ULBP2 expression versus collagen deposition in the human gastric TMAs. Data are expressed as mean ± SEM (***p < 0.001).

To identify antigens that are specifically overexpressed in GC, associated with the dense stromal microenvironment and therapeutic potential, we first performed an mRNA microarray analysis of differentially expressed genes between tumor and adjacent normal tissues from 16 patients with GC, revealing that 666 genes were upregulated. By analysing a cross-dataset of single-cell RNA sequencing (ScRNA-seq) data from GSE163558 and GSE206785, we identified 3227 upregulated genes associated with ECM (Fig. 1A). By referencing immune-related genes from the Immunology Database and Analysis Portal (ImmPort), we further revealed 49 immune-related genes specifically associated with GC. A comparison of the three sets identified overlapping genes, *ULBP2* and *IL-18* (Fig. 1B), indicating that they are immune-related proteins upregulated in GC.

ULBP2 is a cell surface molecule anchored to the membrane via a glycosylphosphatidylinositol (GPI) moiety, and previous studies have identified it as a potential immunotherapeutic target [25]. Through gene filtering, normalisation, and principal component analysis of the scRNA-seq data, we identified nine distinct clusters and observed that ULBP2 was significantly overexpressed in epithelial, fibroblast, and immune cells within tumors compared with normal tissues (Supplemental Fig. S1A). Therefore, we focused on ULBP2 for further analysis. Using the Gene Ontology (GO) database for pathway analysis, collagen fibril organisation, extracellular matrix organisation, and extracellular structure organisation related to the ECM were found to be significantly upregulated (Fig. 1C). CAFs are the primary regulators of the ECM or their secretion and remodelling [3]. Further analysis of ULBP2 expression in fibroblasts revealed a correlation with collagen biosynthetic, collagen fibril organisation, ECM-receptor interaction and TGF-β signalling (Fig. 1D; Supplemental Fig. S1B, C).

To further investigate ULBP2 expression in patients with GC, we first analysed 16 mRNA microarray samples and found that ULBP2 was significantly upregulated in tumors compared to normal tissues (Fig. 1E). Consistent with this, the TCGA database demonstrated the upregulation of ULBP2 in GC tissues (Supplemental Fig. S1D). Next, we performed western blotting and found that ULBP2 protein levels were upregulated in GC tumors compared to those in adjacent normal tissues (Fig. 1F). Furthermore, we analysed ULBP2 expression in 62 paired GC and adjacent normal tissues using tissue microarray (TMA)-based immunohistochemistry (IHC). We found that ULBP2 was highly expressed in GC samples (Fig. 1G). Using Kaplan–Meier Plotter Database analysis, we found that high ULBP2 expression was positively correlated with worse OS in patients with GC, indicating that ULBP2 is an independent prognostic factor (Fig. 1H). Clinical GC samples showed significantly elevated collagen levels in tumors with high ULBP2 expression (Fig. 1I, J). Moreover, we examined ULBP2 expression in 20 human tissues using IHC. We detected almost no expression of ULBP2 in these tissues (Supplemental Fig. S1E), demonstrating that ULBP2 was not abundantly expressed in vital organs. These data indicate that ULBP2 is highly expressed in both tumor epithelial cells and fibroblasts, potentially contributing to the dense stromal microenvironment in tumors.

### ULBP2 promotes the malignant phenotype of GC and is a potential therapeutic target

**Fig. 2:**
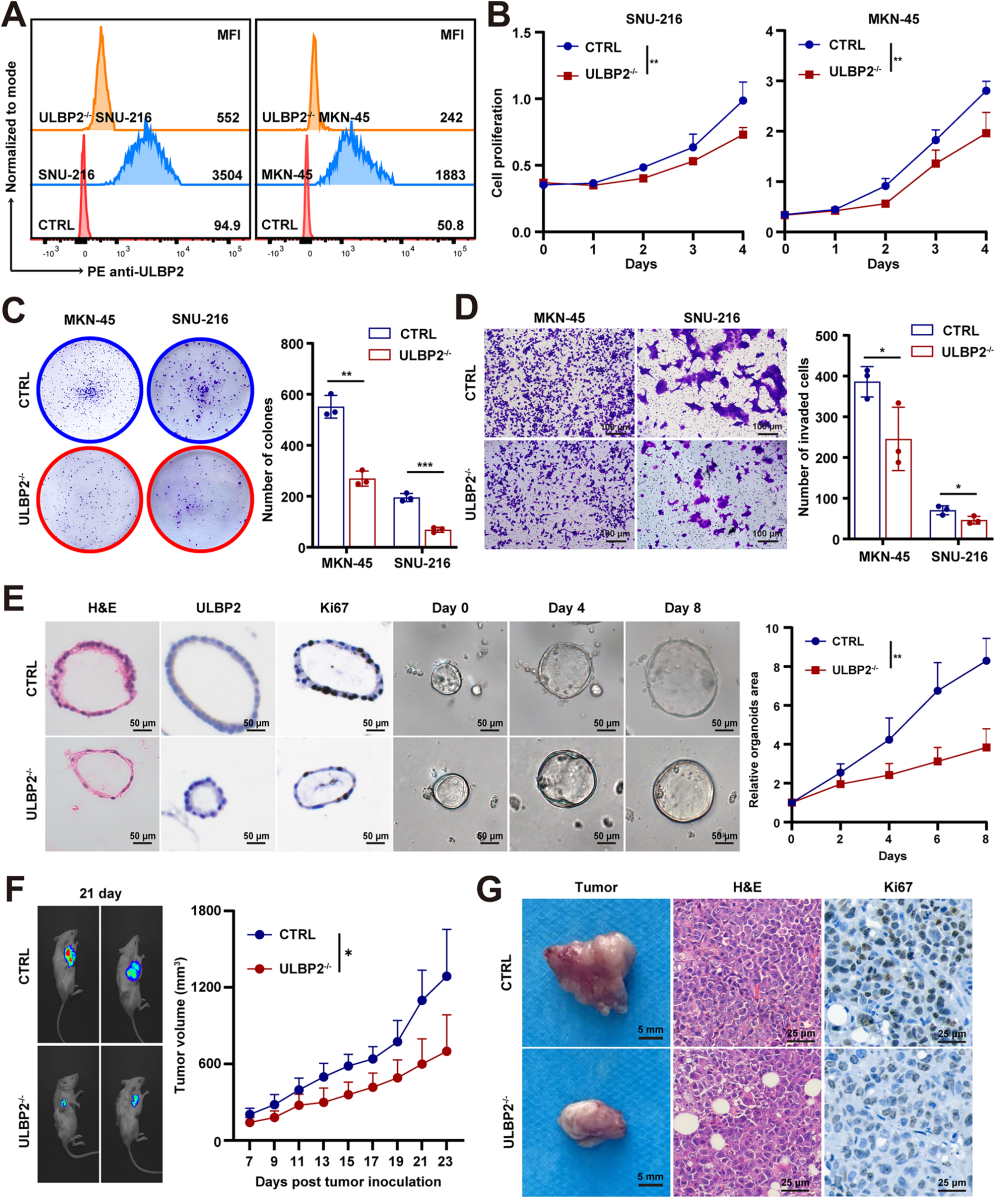
ULBP2 promotes the malignant phenotype of GC. **A** *ULBP2* knockout efficiency in SNU-216 cells and MKN-45 cells assessed by flow cytometry (FCM). **B** Growth curves of ctrl cells and *ULBP2*^−/−^ SNU-216 and MKN-45 cells (n = 3). **C** Colony formation assay and the statistical results of ctrl cells and *ULBP2*^−/−^ SNU-216 and MKN-45 cells (n = 3). **D** Transwell invasion assay of ctrl cells and *ULBP2*^−/−^ SNU-216 and MKN-45 cells (n = 3). **E** Representative images and growth kinetics of ctrl organoids and *ULBP2*^−/−^ organoids (n = 3). **F** Tumor growth curves of NSG mice carrying wild-type, *ULBP2*^−/−^ MKN-45 cell line-derived xenografts (CDX) (n = 5). **G** Representative images of tumors, H&E staining, and Ki-67 IHC staining (n = 3). Data are expressed as mean ± SEM (*p < 0.05, **p < 0.01, ***p < 0.001).

To explore the role of ULBP2 in GC, we identified two GC cell lines (SNU-216 and MKN-45) with higher ULBP2 expression levels compared to (AGS and HGC-27), as determined by western blotting (Supplemental Fig. S2A). We then knocked out *ULBP2* using CRISPR/Cas9 in SNU-216 and MKN-45 cells, and the efficiency was confirmed by flow cytometry (FCM) and western blotting (Fig. 2A, Supplemental Fig. S2B). Using MTT, colony formation, and Transwell invasion assays, we showed that ULBP2 deficiency significantly reduced cell proliferation and invasion in both SNU-216 and MKN-45 cells (Fig. 2B, D). Additionally, we evaluated the impact of *ULBP2* knockout on migration ability using a wound healing assay, which revealed reduced healing capacity in the *ULBP2* knock out group compared with the control group (Supplemental Fig. S2C). Furthermore, we revealed that *ULBP2* knockout effectively induced apoptosis in both SNU-216 and MKN-45 cells (Supplementary Fig. S2H).

We also engineered cell lines to overexpress *ULBP2* and performed proliferation, colony formation, and invasion assays, which indicated that *ULBP2* overexpression promoted the proliferation and invasion of both AGS and HGC-27 cells (Supplemental Fig. S2D–G). Using GC patient-derived organoids, we showed that *ULBP2* knockout significantly reduced the proliferation of organoids (Fig. 2E). In vivo, *ULBP2* knockout MKN-45 cells exhibited a significantly slower growth rate in the CDX mouse model compared to wild-type cells (Fig. 2F). Histological analysis of the excised tumors using haematoxylin and eosin (H&E) and IHC staining of Ki-67 corroborated these findings (Fig. 2G, Supplemental Fig. S2I).

Taken together, these results suggest that ULBP2 plays an oncogenic role in GC. We identified ULBP2 as a cell surface protein that promotes the malignant phenotype of GC and is a potential therapeutic target.

### ULBP2 activates TGF-β signalling and collagen formation in GC

**Fig. 3:**
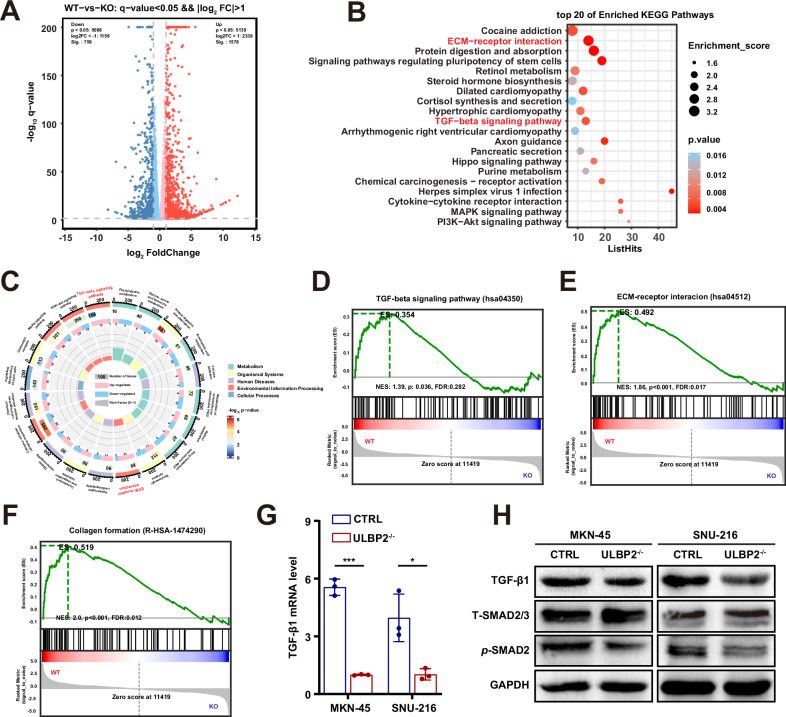
ULBP2 activates TGF-β signalling and collagen formation in GC. **A** Heat map showing differentially expressed genes (DEGs) in *ULBP2*^−/−^ MKN-45 cells compared to wild-type cells. **B** Transcriptome KEGG analysis revealed 20 pathways that *ULBP2* knockout had a significant effect on MKN-45 cells. **C** *ULBP2* knockout exhibited the most pronounced impact on ECM-receptor interaction and TGF-β signalling pathway of GC. **D**–**F** Transcriptome GSEA analysis revealed that the differentially expressed genes were significantly enriched in the TGF-β signalling pathway (**D**), ECM-receptor interaction (**E**), and collagen formation (**F**). **G** mRNA expression levels of TGF-β1 in ctrl cells and *ULBP2*^−/−^ MKN-45 and SNU-216 cells (n = 3). **H** Western blotting of TGF-β1, phosphorylated SMAD2 (*p*-SMAD2), and total SMAD2/3 (T-SMAD2/3) in ctrl cells and *ULBP2*^−/−^ MKN-45 and SNU-216 cells. Data are represented as mean ± SEM (*p < 0.05, ***p < 0.001).

To elucidate the underlying mechanisms of ULBP2 in GC, we performed transcriptomic analysis of *ULBP2* knockout MKN-45 cells and compared them with wild-type cells. A total of 2138 differentially expressed genes (DEGs) were identified, including 1426 genes upregulated and 712 downregulated genes (Fig. 3A). Using the Gene Ontology (GO) and Kyoto Encyclopedia of Genes and Genomes (KEGG) databases for pathway analysis of the DEGs within the transcriptome, we identified 20 pathways that exhibited the lowest *p*-values. Notably, the *ULBP2* knockout had the most pronounced impact on TGF-β signalling and ECM-receptor interaction (Fig. 3B, C; Supplemental Fig. S3A). Furthermore, Gene Set Enrichment Analysis (GSEA) supported these findings by revealing significant enrichment of TGF-β Signalling, ECM-receptor interaction, and collagen formation pathways (Fig. 3D–F).

To further validate these findings, we first performed real-time quantitative reverse transcription PCR (qRT-PCR) to determine the transcription levels of the TGF-β signalling pathway genes. We found that the mRNA expression of TGF-β1 was significantly reduced in *ULBP2* knockout MKN-45 and SNU-216 cells compared with wild-type cells (Fig. 3G). We also performed western blotting and found that TGF-β1 and phosphorylated SMAD2 protein levels were downregulated in *ULBP2* knockout cells (Fig. 3H). TGF-β signalling, a critical regulator of diverse biological processes, is generally accepted for its dual role in tumorigenesis, acting as a tumor suppressor in early stages while promoting tumor progression at later stages [26]. TGF-β stimulates fibroblasts, transforming them into CAFs, which regulate the production and remodelling of ECM components to establish a dense stromal microenvironment [27]. Moreover, TGF-β can facilitate the progression of epithelial-derived tumors through the induction of EMT [28]. Thus, we evaluated the expression of EMT-associated factors and found that the ULBP2 knockout upregulated E-cadherin expression and downregulated vimentin expression (Supplemental Fig. S3B, C).

These results collectively indicate that the ULBP2 may promote GC proliferation and collagen deposition via the TGF-β signalling pathway. We hypothesised that ULBP2 overexpression might activate the TGF-β signalling pathway, thereby upregulating TGF-β expression and promoting CAF activation and stromal deposition.

### ULBP2 promotes CAF activation via TGF-β signalling in GC

**Fig. 4:**
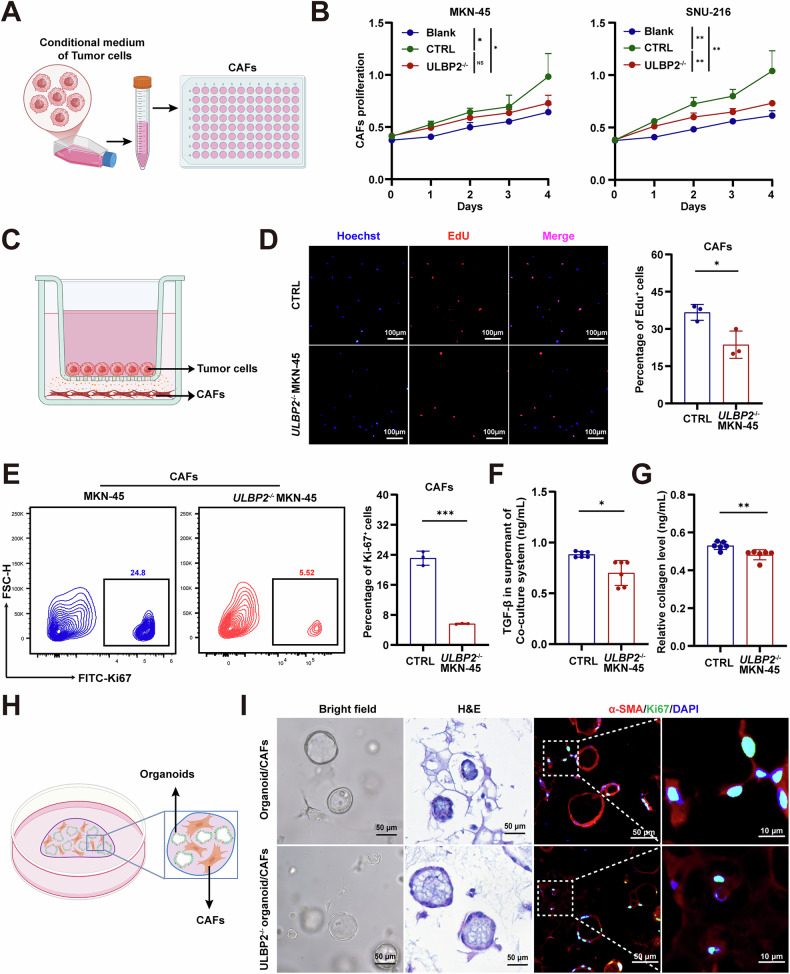
ULBP2 promotes CAF activation via TGF-β signalling in GC. **A** Diagram of the experimental procedure. **B** Growth curves of CAFs cultured with supernatants from ctrl cells and *ULBP2*^−/−^ SNU-216 and MKN-45 cells (n = 3). **C** The diagram of co-culture system between CAFs and GC cells. **D** EdU assay showing that the CAF proliferation was significantly reduced in the co-culture system of *ULBP2* knockout MKN-45 cells compared with wild-type cells (n = 3). **E** Frequency of Ki-67 positive cells in the co-culture system, as determined by FCM (n = 3). **F**, **G** Relative levels of TGF-β1 (**F**) and collagen (**G**) content in the co-culture system (n = 6). **H** Diagram of the co-culture system between CAFs and GC organoids. **I** Representative bright field, H&E, and α-SMA staining images of the CAFs and organoid co-culture system. Data are represented as mean ± SEM (ns, nonsignificant, *p < 0.05, **p < 0.01, ***p < 0.001).

To investigate the impact of ULBP2 on CAF activation, tumor cell culture supernatants were collected and added to the CAF culture system (Fig. 4A). The results demonstrated that CAF proliferation was significantly reduced when cells were exposed to supernatants from *ULBP2* knockout MKN-45 and SNU-216 cells, compared to wild-type cells (Fig. 4B), while *ULBP2* overexpression significantly enhanced CAF proliferation (Supplemental Fig. S4A). Next, we established another co-culture system in which tumor cells were cultured in the upper chamber of a Transwell chamber, and CAFs were cultured in the lower chamber (Fig. 4C). Using EdU proliferation and FCM assays, we showed that ULBP2 deficiency in both MKN-45 and SNU-216 cells lead to a significant reduction in CAF proliferation in this co-culture system (Fig. 4D, E; Supplemental Fig. S4B, C). Moreover, TGF-β and collagen protein levels were markedly decreased in the co-culture system with *ULBP2* knockout MKN-45 and SNU-216 cells compared to wild-type cells (Fig. 4F, G; Supplemental Fig. S4D, E). We also conducted Transwell invasion assays in the CAF co-culture system (Supplemental Fig. S4F). *ULBP2* knockout significantly reduced the number of CAFs that penetrated the lower chamber (Supplementary Fig. S4G).

To further explore the role of ULBP2 in CAF activation, we developed organoid and CAF co-culture systems, demonstrating that *ULBP2* knockout organoids significantly suppressed α-SMA activation compared to wild-type organoids (Fig. 4H, I). Altogether, these results indicate that ULBP2 overexpression activates the TGF-β signalling pathway and upregulates TGF-β expression, thereby promoting CAF activation and ECM remodelling, and contributing to the formation of a dense stromal microenvironment.

### ULBP2 CAR-T cells eliminate GC cells and organoids

To develop CAR-T cell-based therapy for GC, we first generated a mouse-derived monoclonal antibody (mAb) targeting ULBP2. We then construct chimeric antigen receptor-expressing T (CAR-T) cells based on the novel anti-ULBP2 mAb, and evaluated the anticancer efficacy of ULBP2 CAR-T cells.

**Fig. 5:**
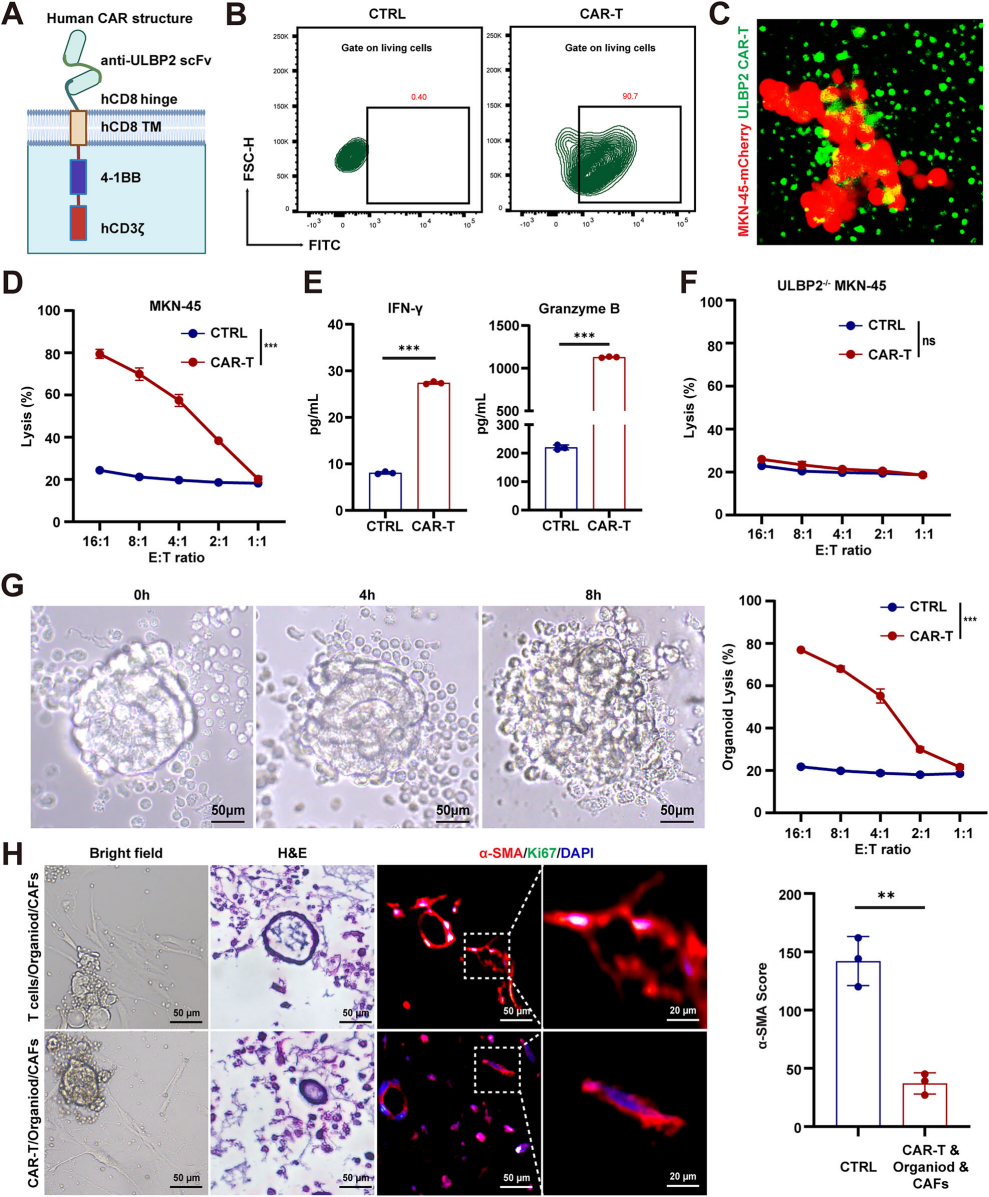
ULBP2 CAR-T cells specifically eliminate GC cells and organoids. **A** Schematic diagram of ULBP2 CAR. **B** FCM showing the transduction efficiency of CAR-T cells. **C** IF analysis of the binding of ULBP2 CAR-T cells (green) to MKN-45-mCherry cells (red). **D** Killing efficiency of MKN-45 cells by ULBP2 CAR-T cells, determined by LDH-based cytotoxicity assay (n = 3). **E** Protein levels of IFN-γ and granzyme B in the culture supernatant (n = 3). **F** Killing efficiency of *ULBP2*^−/−^ MKN-45 cells by ULBP2 CAR-T cells (n = 3). **G** Representative images of GC organoids and killing efficiency of organoids by ULBP2 CAR-T cells (n = 3). **H** Representative bright field, H&E, and α-SMA staining images of ULBP2 CAR-T cells, CAFs, and organoid co-culture system, along with quantification of α-SMA positive area (n = 3). Data are represented as mean ± SEM (ns, nonsignificant; ***p < 0.001).

We constructed a second-generation CAR consisting of a single-chain variable fragment (scFv) derived from the anti-ULBP2 mAb, human CD8 hinge and transmembrane domains, a human 4-1BB co-stimulatory domain, and a CD3ζ signalling domain (Fig. 5A). FCM analysis revealed a transduction efficiency of 90.7% for T cells transduced with ULBP2 CAR lentivirus (Fig. 5B). The immunofluorescence (IF) assay revealed that ULBP2 CAR-T cells expressing green fluorescent protein (GFP) could specifically recognize MKN-45-mCherry cells expressing red fluorescent protein (mCherry) (Fig. 5C). Additionally, ULBP2 CAR-T cells effectively lysed wild-type MKN-45 cells, accompanied by elevated concentrations of IFN-γ and granzyme B in the culture medium compared to control T cells (Fig. 5D, E; Supplemental Fig. S5A). Similarly, ULBP2 CAR-T cells effectively lysed SNU-216 cells, which express high levels of ULBP2 and AGS cells engineered to overexpress ULBP2 (Supplemental Fig. S5B, C). A low killing activity of ULBP2 CAR-T cells was observed in *ULBP2* knockout MKN-45 and SNU-216 cells (Fig. 5F; Supplemental Fig. S5D).

We further evaluated the anti-tumor efficacy of ULBP2 CAR-T cells using GC organoids. ULBP2 CAR-T cells effectively lysed the organoids, demonstrating their potent ULBP2-dependent cytotoxicity in a clinically relevant in vitro model (Fig. 5G). We also established a co-culture system consisting of ULBP2 CAR-T cells, CAFs, and organoids and found that ULBP2 CAR-T cells not only effectively eliminated organoids, but also suppressed CAF activation within the co-culture environment (Fig. 5H). Collectively, these results highlight the specificity and efficacy of ULBP2 CAR-T cells and provide a promising therapeutic strategy for GC.

### ULBP2 CAR-T cells alone or in combination with PD-1 blockade suppress GC growth and prolong survival

**Fig. 6:**
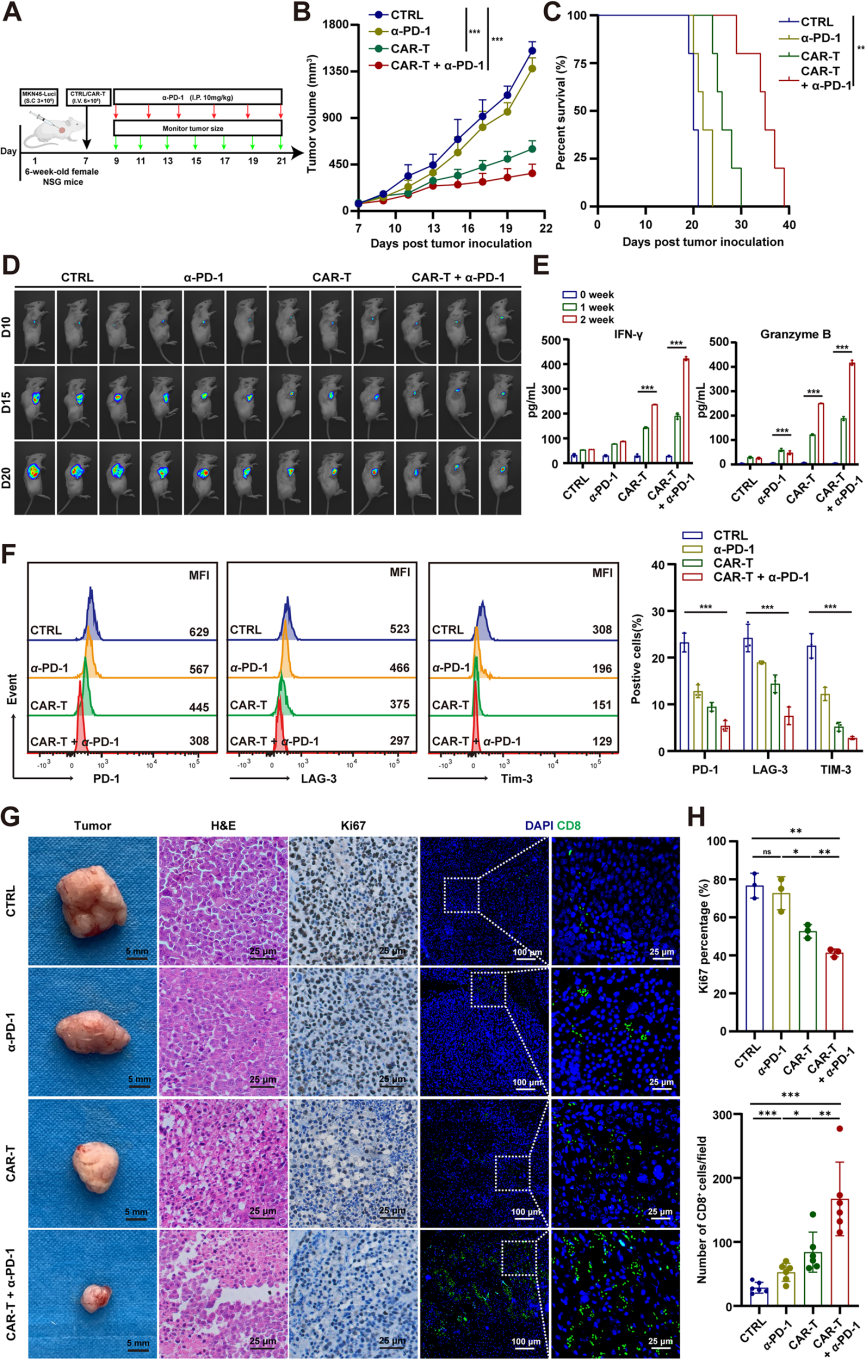
ULBP2 CAR-T cells alone or in combination with PD-1 blockade suppress GC growth and prolong survival. **A** Diagram of the experimental procedure. **B**, **C** Tumor growth (**B**) and survival (**C**) curves of the CDX mice treated with ULBP2 CAR-T, anti-PD-1, or ULBP2 CAR-T cells plus anti-PD-1 (n = 5). **D** Bioluminescent imaging of the CDX mice at different days post tumor inoculation. **E** Protein levels of IFN-γ and granzyme B in the serum of CDX mice at different weeks post tumor inoculation detected by ELISA (n = 3). **F** Protein levels of PD-1, LAG-3, and TIM-3 in tumor-infiltrating lymphocytes at day 21 post tumor inoculation in the CDX mice that received different treatments, as shown by FCM (n = 3). **G** Representative images of tumors, H&E staining, Ki-67 IHC staining, and CD8 IF staining in the CDX mice that received different treatments. **H** Quantification of Ki-67 (n = 3) and CD8^+^T (n = 6) cells in the CDX at day 21 post tumor inoculation. Data are represented as mean ± SEM (*p < 0.05, **p < 0.01, ***p < 0.001).

To examine the effects of ULBP2 CAR-T cells in vivo and the potential to enhance the efficacy of anti-PD-1 therapy, we first generated a CDX model using NSG mice inoculated with MKN-45-ULBP2-T2A-Luc cells. When the average tumor size reached 90 mm^3^, CDX mice were infused with ULBP2 CAR-T cells. One day later, mice in the combination group were treated with anti-PD-1 antibodies every 5 days. Un-transduced human T cells were used as a negative control and anti-PD-1 monotherapy (10 mg/kg, intraperitoneal injection) served as a positive control. Tumor growth and body weight were monitored throughout the course of the experiment (Fig. 6A). We found that ULBP2 CAR-T cells potently curbed tumor growth and prolonged survival compared to un-transduced T cells and anti-PD-1 treatment groups. The anti-tumor effect of ULBP2 CAR-T cells was further significantly enhanced by co-treatment with anti-PD-1 (Fig. 6B–D; Supplemental Fig. S6A–C). Peripheral blood was collected on days 7 and 14, and CAR-T cells were consistently detected with robust expansion, indicating the successful engraftment of CAR-T cells (CD45^+^CD3^+^CD4^+^CD8^+^) in NSG mice (Supplemental Fig. S6D, E). Higher concentrations of IFN-γ and granzyme B were detected in the serum of ULBP2 CAR-T cells treated mice than control mice, indicative of an activated immune response of the infused CAR-T cells (Fig. 6E). We measured cytokines including IL-6, IL-1β and TNF-α and found that CAR-T cell therapy did not induce severe cytokine release syndrome (CRS) (Supplemental Fig. S6F). The expression of molecules associated with T cell exhaustion [29], such as PD-1, lymphocyte activation gene 3 (LAG-3), and T cell immunoglobulin, and mucin domain-containing 3 (TIM-3), decreased in tumor-infiltrating CAR-T cells compared with that in un-transduced T cells, especially in the presence of anti-PD-1 mAb (Fig. 6F). Additionally, we performed haematoxylin and eosin (H&E) and IHC staining of Ki-67 and IF staining of CD8 in the excised tumors. The results were consistent with the above findings (Fig. 6G, H). Notably, no significant changes in body weight were observed in any of the treatment groups, suggesting the safety of ULBP2 CAR-T therapy (Supplemental Fig. S6B).

**Fig. 7:**
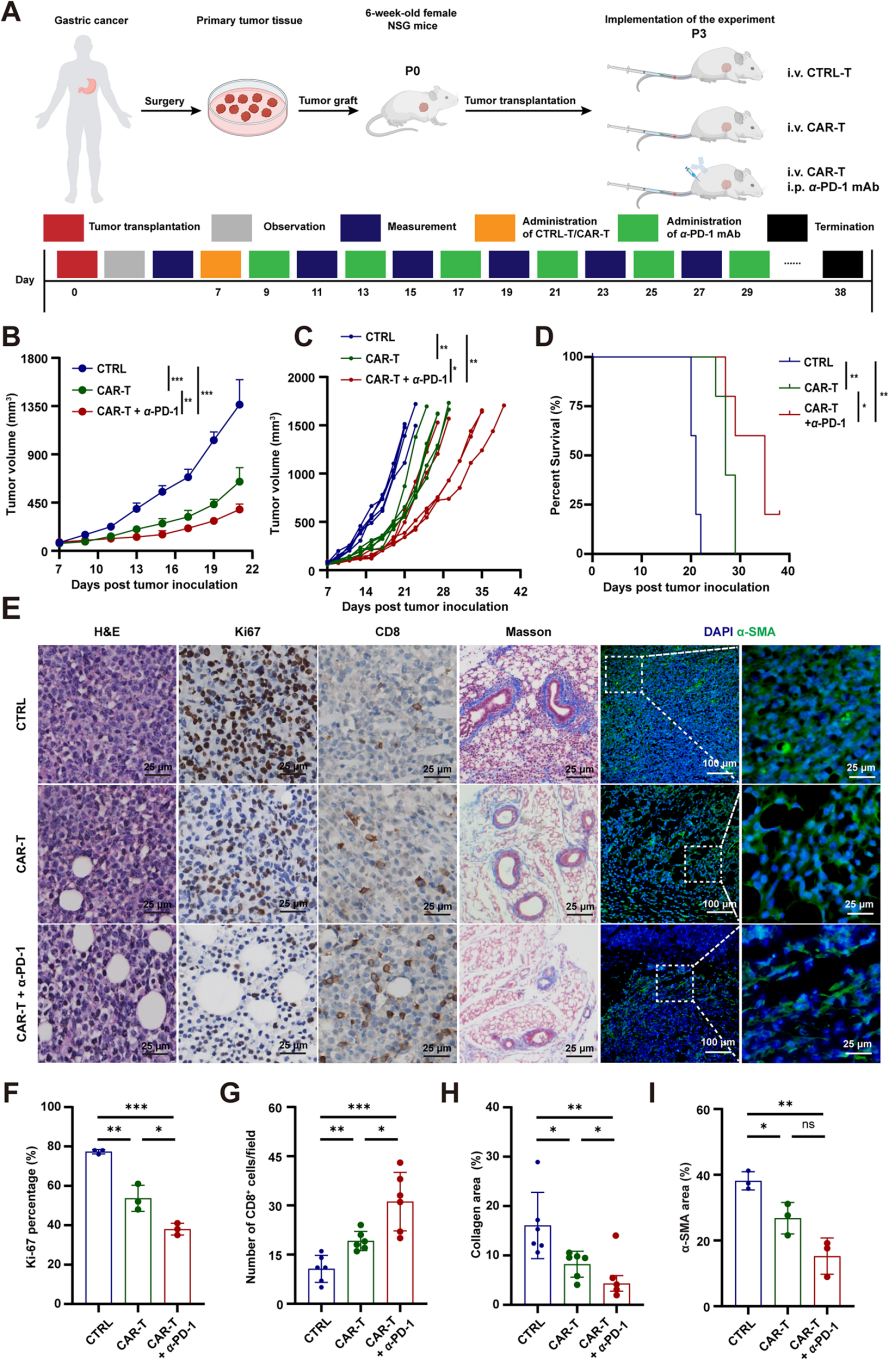
ULBP2 CAR-T cells alone or in combination with anti-PD-1 improves survival of NSG mice bearing patient-derived xenografts. **A** Diagram of the experimental procedure. **B**–**D** Tumor growth **B** and survival curves (**C**, **D**) of mice bearing the PDX from a patient treated with ULBP2 CAR-T or ctrl cells alone, or ULBP2 CAR-T cells plus anti-PD-1 (n = 5). **E** Representative images of H&E, Ki-67 IHC, CD8 IHC, Masson, and α-SMA IF staining in the PDX mice that received different treatments. **F**–**I** Quantification of Ki-67 **F** (n = 3), CD8^+^T cells **G** (n = 6), collagen area **H** (n = 6), and α-SMA area (n = 3) in the PDX at day 21 post tumor inoculation. Data are represented as mean ± SEM (*p < 0.05, **p < 0.01, ***p < 0.001).

To further evaluate the efficacy of ULBP2 CAR-T cells alone and in combination with anti-PD-1 antibodies against GC, we established a GC PDX model [30]. Fresh tumor specimens were obtained from a patient with GC (the clinical characteristics was shown in Table S1) and serially transplanted into 6-week-old female NSG mice to produce sufficient amounts of primary tumor tissue. The third generation of serially transplanted mice were used for the experiments when the average tumor size reached 90 mm^3^ (Fig. 7A). We observed that the growth of PDX was significantly hindered by ULBP2 CAR-T cells alone compared to that of un-transduced T cells, and was further impeded by the combination with anti-PD-1 (Fig. 7B; Supplemental Fig. S7A). More importantly, the combination therapy markedly improved survival.

While all five mice in each of the other groups died within 29 days, one out of five mice in the combination therapy group survived beyond 38 days (Fig. 7C, D). In line with these studies, we found that the combination therapy strongly inhibited cell proliferation, as shown by H&E and IHC staining (Fig. 7E, F). IHC staining revealed a significant increase in the number of CD8^+^ T cells in the ULBP2 CAR-T cell-treated mice compared with that in the control mice, and co-treatment with anti-PD-1 further enhanced the infiltration of cytotoxic T cells into the tumor (Fig. 7E, G). Using Masson’s trichrome and IF assays, we showed that ULBP2 CAR-T cell treatment reduced stromal deposition and CAF activation (Fig. 7H, I). Moreover, none of the ULBP2 CAR-T cell treatments caused significant changes in the body weight of the mice, indicating the safety and tolerability of ULBP2 CAR-T cells alone and in combination with anti-PD-1 mAb (Supplemental Fig. S7B). Therefore, ULBP2 CAR-T cells alone strongly inhibit GC growth and prolong survival, and the anti-tumor effects are further augmented when combined with PD-1 blockade.

## Discussion

**Fig. 8:**
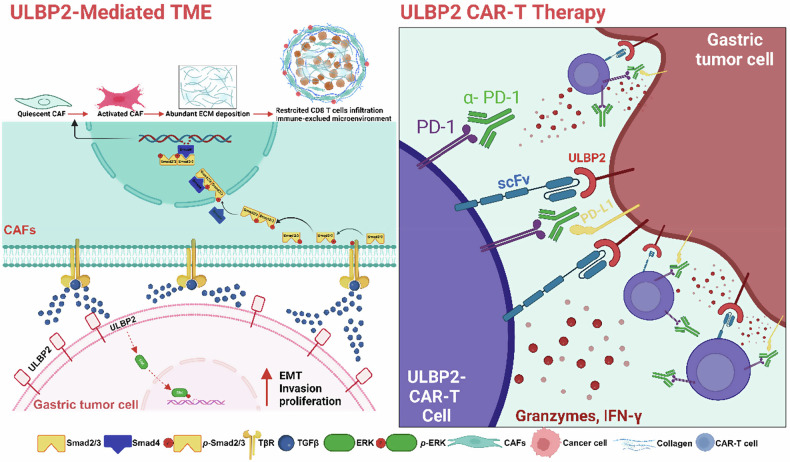
ULBP2 CAR-T cells potentiate anti-PD-1 efficacy in GC. ULBP2 overexpression promotes TGF-β signalling, driving CAF activation, leading to a dense stromal microenvironment. Moreover, targeting ULBP2 effectively reduces stromal deposition, stimulates CAR-T cell infiltration, and augments the antitumor efficacy of PD-1 blockade therapy. This graphical abstract was created with BioRender.com (License #NP28FJISA5, 2025).

Despite the significant therapeutic progress in immunotherapy for GC, many challenges still remain including the lack of effective therapeutic targets, and the prevalence of primary or acquired resistance in most patients. Here, we identified ULBP2 as a potential therapeutic target for GC and developed ULBP2 CAR-T cells. We demonstrated the efficacy of ULBP2 CAR-T cells alone, and particularly in combination with PD-1 blockade, to inhibit tumor growth and promote survival, using GC cell lines and organoids, as well as CDX and PDX models. In addition, ULBP2 overexpression promotes TGF-β signalling, driving CAF activation, leading to a dense stromal microenvironment. Targeting ULBP2 effectively reduced stromal deposition, stimulated CAR-T cell infiltration, and augmented the antitumor efficacy of PD-1 blockade therapy (Fig. 8). Our findings suggest a promising new therapeutic strategy for the treatment of GC.

CAR-T cell therapy has led to breakthroughs in the treatment of gastrointestinal (GI) tract cancers. CLDN18.2 CAR-T cells have demonstrated promising efficacy against CLDN18.2-positive digestive cancers, particularly in GC [10]. CYAD-101, a NKG2D-based, non-gene-edited allogeneic CAR-T cell line, has shown the ability to sustain CAR-directed anti-tumor activity without inducing graft-versus-host disease (GvHD) [31, 32]. However, a major limitation of CAR-T cell therapy is the lack of safely targetable cancer antigens on the cell surface [33]. Here, we identified ULBP2 as an immune-related cell surface protein that is overexpressed in GC, but absent in adjacent normal tissues.

Previous studies have shown that ULBP2 is expressed in many types of human cancers, including breast cancer [34], pancreatic cancer [23], colon cancer [24], and ovarian cancer [35, 36]. In our study, we further validated ULBP2 overexpression in GC using western blotting, qRT-PCR, and IHC staining. Additionally, we found that high ULBP2 expression serves as an independent prognostic factor for poor OS in GC. The high positivity rate and prognostic relevance of ULBP2 indicates that it could be a promising alternative therapeutic target in GC.

ULBP2 is a cell surface molecule that binds to the NKG2D receptor and exists either as a membrane-bound form via a glycosylphosphatidylinositol (GPI) anchor or as a secreted protein [37]. ULBP2 is frequently expressed in tumor cells where it activates natural killer (NK) cells through NKG2D receptor engagement, enabling NK cell-mediated tumor cell cytotoxicity [38]. However, tumors employ various mechanisms to inhibit the NKG2D/NKG2DL axis, thereby facilitating immune evasion. Notably, the proportion of NKG2D-positive NK cells is reduced in pancreatic, gastric, and colorectal cancers, correlating with poor prognosis in these malignancies [39]. Soluble ULBP2 derived from tumor cells impairs immune surveillance by inhibiting NKG2D expression and NK cell activity [40, 41]. Similarly, TGF-β is an important component in the tumor microenvironment (TME) that promotes the occurrence and growth of tumor cells and has immunosuppressive activity, particularly by downregulating the expression of NKG2D on the surface of NK cells [42, 43]. Our findings revealed that *ULBP2* knockout significantly suppresses the proliferation and invasion of GC cells, both in vitro and in vivo. Transcriptomic analysis using the KEGG data identifies the TGF-β signalling pathway and ECM−receptor interaction as the most significantly affected pathways. Furthermore, we demonstrate that ULBP2 enhances immunosuppression in GC by activating CAFs and promoting collagen deposition via the TGF-β signalling pathway. These findings highlight the oncogenic role of ULBP2 and its potential as a therapeutic target in cancer.

The overexpression of ULBP2 in tumors and its pleiotropic roles in cancer progression make it an attractive therapeutic target [25]. Previous studies developed NKG2D-based CAR-T and CAR-NK cells to target ligands in cancer and senescent cells [44–47]. Here we engineered a second-generation ULBP2 CAR-T cells using a novel anti-ULBP2 monoclonal antibody (mAb). These CAR-T cells effectively killed ULBP2-expressing cells and GC patient-derived organoids in vitro. ULBP2 CAR-T cells alone or in combination with PD-1 mAb significantly suppressed tumor growth in MKN-45-ULBP2-T2A-Luc derived CDX models. Currently, patient-derived xenograft (PDX) models are widely regarded as the most clinically relevant in vivo cancer models due to their preservation of key tumor characteristics and high translational fidelity to patient drug responses [30, 48, 49]. In our study, we demonstrated that treatment with ULBP2 CAR-T cells, particularly in combination with anti-PD-1 antibodies, markedly prolonged the survival of PDX mice. Additionally, ULBP2 CAR-T cells exhibited enhanced resistance to T cell exhaustion compared with un-transduced T cells, especially when combined with anti-PD-1 mAb. Therefore, combination therapy consisting of ULBP2 CAR-T cells and anti-PD-1 could be a promising strategy for GC treatment.

In conclusion, we demonstrate that ULBP2 activates the TGF-β signalling pathway, promoting collagen deposition and CAF activation, which collectively fosters a dense stromal microenvironment in GC. The combination of ULBP2 CAR-T cells and anti-PD-1 mAb significantly inhibits tumor growth and improves survival in preclinical models. Our findings highlight the therapeutic potential of targeting ULBP2 in combination strategies to improve the clinical outcomes of patients with GC.

## Material and methods

### Cells and culture conditions

Human GC cell lines SNU-216, MKN-45, HGC-27, and AGS were obtained from the Chinese Academy of Medical Sciences (China). HEK-293T cells were obtained from the American Type Culture Collection (ATCC). All cell lines were authenticated by short tandem repeat (STR) DNA fingerprinting and confirmed to be free from mycoplasma contamination. SNU-216, HGC-27, AGS, and HEK-293T cells were cultured in DMEM supplemented with 10% foetal bovine serum, 100 U/mL penicillin, and 100 μg/mL streptomycin. MKN-45 cells were cultured in RPMI-1640 medium supplemented with 10% FBS, 100 U/mL penicillin, and 100 μg/mL streptomycin. CD3^+^ T cells were enriched from human peripheral blood mononuclear cells (hPBMCs) using the EasySep Human T Cell Isolation Kit (STEMCELL, 17951) and cultured in RPMI-1640 medium supplemented with 10% FBS, 100 U/mL penicillin, 100 μg/mL streptomycin, and 10 ng/mL recombinant human IL-2 (STEMCELL, 78036). All cells were maintained at 37 °C in a humidified incubator with 5% CO_2_.

### Animals

M-NSG mice (NOD-*Prkdc*^scid^*Il2rg*^em1^/*Smoc*) lacking mature T, B, and NK cells were obtained from Shanghai Model Organisms Center, Inc. C57BL/6 mice were provided by the Lanzhou Veterinary Research Institute. All animals were female, aged 6–8 weeks, maintained under specific pathogen-free (SPF) conditions, and randomly assigned to experimental groups using a random number table. Animal care and experiments were conducted in accordance with the Guide for the Care and Use of Laboratory Animals from the National Institute of Health.

### Human GC specimens

Tissue microarrays (TMAs) comprising 62 pairs of GC and adjacent normal tissues were prepared as formalin-fixed, paraffin-embedded blocks, and analysed using immunohistochemistry (IHC). Additionally, 20 normal tissues were included for IHC staining of ULBP2 (Proteintech, 13133-1-AP), and six pairs of GC and adjacent normal tissues were used for gene expression analysis by western blotting.

### Bioinformatics analysis of gene expression profiles in GC

Single-cell sequencing datasets (GSE163558 and GSE206785) and corresponding clinical information were obtained from the Gene Expression Omnibus database. The major cell types in GC were annotated based on cellmarker2.0, while pathway-specific gene sets were curated from The Molecular Signatures Database (MsigDB, https://www.gsea-msigdb.org/gsea/msigdb). Immune-related genes in human cancers were retrieved from the Immunology Database and Analysis Portal (http://www.immport.org/). The pathway activity and gene scores were computed using the add-module score function.

### Generation of anti-ULBP2 monoclonal antibody

To generate monoclonal antibodies against human ULBP2, 6-week-old C57BL/6 mice were immunised three times via subcutaneous multipoint injections with 1 × 10^7^ HEK-293T cells stably expressing ULBP2. Serum samples were collected via retroorbital bleeding to monitor antibody titres. Five days before splenectomy for monoclonal antibody generation, the mice were boosted with a subcutaneous multipoint injection of 2 × 10^6^ HEK-293T-ULBP2 cells. Monoclonal antibodies were subsequently prepared using Microfluid-based Antibody Tech (MBAT, Huazhitiancheng Inc.).

### Generation of ULBP2 CAR

The amino acid sequence of the single-chain variable fragment (scFv) specific to human ULBP2 was derived from the variable regions of heavy and light chains of the generated anti-ULBP2 monoclonal antibody. A second-generation CAR (Chimeric Antigen Receptor) was engineered, consisting of anti-ULBP2 scFv, human CD8-derived hinge and transmembrane, and human 4-1BB co-stimulatory and CD3ζ signalling domains.

### Lentiviral vector production

HEK-293T cells were used for lentiviral vector production. To generate stable cell lines, including HGC-27-ULBP2-T2A-GFP, AGS-ULBP2-T2A-GFP, MKN-45-ULBP2-T2A-Luc, as well as *ULBP2*^*−/−*^ MKN-45 and *ULBP2*^*−/−*^ SNU-216, pseudotyped lentiviruses were produced by co-transfecting HEK-293T cells in a 3.5-cm dish with 1 μg pLenti-EF1α-ULBP2-T2A-GFP, 1 μg pMD2G, and 0.5 μg psPAX2 into HEK-293T cells in a 3.5-cm dish. After 48 h post-transfection, lentivirus-containing supernatants were collected, filtered through a 0.45 μm non-pyrogenic filter, and immediately used to transduce target cells in the presence of polybrene. Transduced cells were selected with 2 μg/mL puromycin for 5–7 days to establish stable cell lines. To generate *ULBP2* knockout SNU-216 and MKN-45 cell lines, pLenti-CRISPR-V2, pSPAX2, and pMD2G were used. Transduction and selection were conducted as previously described. Guide RNA (gRNA) sequences used for the CRISPR/Cas9-mediated *ULBP2* knockout are listed in Table S2.

Lentivirus production for CAR-T cell transduction was performed using HEK-293T cells (6 × 10^6^ cells per 10-cm dish). After 18 h, cells were transfected with 7 μg Lenti-CAR plasmid, 5 μg psPAX2 plasmid, and 3.5 μg pMD2G plasmid, using 30 μL Lipofectamine 2000 in 500 μL opti-MEM medium. After 6 h, fresh DMEM containing 10% FBS was added to each well. At 60 h post-transfection, the cell culture supernatants were collected, centrifuged at 1000 × *g* for 10 minutes at 4 °C to remove cellular debris, and filtered through 0.45-μm low protein-binding membranes. The lentiviruses were concentrated in DMEM containing 10% FBS and 1% bovine serum albumin.

### Preparation of ULBP2 CAR-T cells

T cells were purified from hPBMCs using the EasySep Human T Cell Isolation Kit (STEMCELL, 17951) and stimulated with CD3/CD28 T cell activator Dynabeads (Gibco, 11131D) at a bead-to-cell ratio of 1:2 and IL-2 (10 ng/mL). After 24 h, lentiviral transduction was performed by centrifugation at 1000 × *g* and 32 °C for 90 minutes in the presence of 4.4 μg/mL polybrene. After 10 h, the viral supernatant was replaced with fresh culture medium. T cells were expanded by replacing half of the culture medium with fresh medium every 2 days. After 6 days, the Dynabeads were removed, and T cells were allowed to expand for an additional 3 days. Transduction efficiency was evaluated by FCM, and ULBP2 CAR-T cells were subsequently used for in vitro assays or adoptively transferred into mice.

### Cytotoxicity assays

The cytotoxicity of ULBP2 CAR-T cells was determined using standard lactate dehydrogenase (Promega, J2381). A 96-well plate was taken and 100 μL cell suspension added (cell density is 1 × 10^5^ /mL); 100 μL test substance were added to each well. For the natural release control, 100 μL cell suspension and 100 μL of culture medium were added. For the maximum release control, 100 μL of cell suspension and 100 μL of 0.2% TritonX-100 were added. Each condition was tested in triplicate, and the plates were incubated at 37 °C with 5% CO_2_ for 8 h.

After incubation, the 96-well plates were centrifuged at 250 × *g* for 5 min, and 50 μL of the suspension was transferred from each well to a new 96-well plates. Then, 50 μL of CytoTox 96 reagent was added to each well. After 30 min reaction at room temperature, 50 μL of stop solution was added to terminate the reaction, and the relative luminescence units (RLUs) of each well were measured. Cytotoxicity was calculated using the following formula: cytotoxicity (%) = [RLU of reaction - RLU of natural release/RLU of maximum release - RLU of natural release] × 100%.

### Organoid culture

GC organoids were generated according to established protocols. In brief, tumor tissues were collected from patients with GC (the clinical characteristics was shown in Table S1), washed with human washing medium (DMEM/F 12 supplemented with 2 mM glutamine and 10% FBS), minced with scissors, and enzymatically digested using 0.1 mg/mL collagenase IV at 37 °C for 30 min. The digestion was halted by adding human washing medium and the digested tumor tissues were filtered through a 100-μm cell strainer. The retained cell clusters were pelleted at 300 g for 5 min, resuspended in 50% Matrigel (R&D, BME001-05)/organoid culture medium (BioGenous, K2179-GC), and plated as 50 μL drops in the centre of pre-warmed 24-well plates. The drop was allowed to solidify by incubation at 37 °C and 5% CO_2_ for 30 min. Once solidified, organoid culture medium (0.5 mL) was added, and the medium was replaced every 3–4 days.

### Co-culture of organoids and fibroblasts

To establish a co-culture system, organoids were resuspended and cultured in a healthy state. After dissociation from Matrigel, organoids were washed several times with pre-cooled phosphate buffered saline (PBS) to remove most of the matrix components and then resuspended in the co-culture medium following cell counting. Adherent CAFs were digested with trypsin and resuspended in the co-culture medium after counting. Counted organoids and CAFs were then mixed with Matrigel and co-culture medium and plated as 50 μL droplets in the centre of pre-warmed 24-well plates. The droplets were incubated at 37 °C and 5% CO_2_ for 30 min to allow solidification. After solidification, the co-culture medium (0.5 mL) was added, with medium changes every 2–3 days. After 6 days, the co-culture was established, and the medium, along with organoids/CAFs, was collected for further experiments.

### Mouse tumor models

The cell-derived xenograft (CDX) model was established using MKN-45 cells stably expressing ULBP2 and firefly luciferase (MKN-45-ULBP2-T2A-Luc). Cells were suspended in 100 μL of PBS and mixed with 100 μL of Matrigel (Corning, 354234), and injected subcutaneously (3 × 10^6^ per mouse) into 6-week-old NSG mice. Bioluminescence imaging was conducted at designated time points using a VISQUE in vivo Smart-L. Tumors were harvested at the experimental endpoint.

To establish the PDX patient-derived xenograft (PDX) model, fresh tumor tissues were obtained from patients with GC (the clinical characteristics were shown in Table S1), dissected into small pieces (approximately 10 mm^3^), and subcutaneously injected into 6-week-old NSG mice for serial transplantation. For CAR-T cell therapy studies, mice received adoptive transfer of transduced ULBP2 CAR-T cells (6 × 10^6^ per mouse, intravenous injection) when the average tumor size reached 90 mm^3^, and the combination therapy group received anti-PD-1 (10 mg/kg, intraperitoneal injection) every 5 days. Un-transduced human T cells were used as a negative control and anti-PD-1 monotherapy (10 mg/kg, intraperitoneal injection) served as a positive control. Tumor volume was measured using a digital calliper, and body weight was monitored every other day. The tumor volume was calculated using the following formula: tumor volume = (longer diameter) × (shorter diameter)^2^/2. The experimental endpoint was defined as either death or a tumor size of 1500 mm^3^. Tumor tissues were collected at the endpoint, fixed in formalin, and embedded in paraffin for H&E, IHC, and IF staining. Blinding was implemented throughout the study to minimize bias. Investigators responsible for administering treatments and assessing outcomes were blinded to group allocations during all experimental procedures. All animal procedures were performed using five mice per group. The group size was determined according to published studies in this field and in accordance with the 3 R principles of animal research ethics.

### Flow cytometry analysis

To analyse cells from the tumor models, tumors isolated from mice were minced with scissors and digested with collagenase IV to obtain single-cell suspensions. The resulting cell slurry was passed through a 70-μm filter and transferred into a 15-mL centrifuge tube. After centrifugation at 300 × *g* for 5 min, the supernatant was discarded and the pellet was resuspended in 2 mL of freshly prepared 45% Percoll solution. The suspension was centrifuged at 350 × *g* for 7 min. Erythrocytes were lysed using Red Blood Cell Lysis Buffer, and the remaining cells were resuspended in PBS containing 2% FBS, stained with specific antibodies for 30 min in the dark, and analysed using flow cytometry. The following antibodies were used: FITC anti-human CD3 (Biolegend, 100204), PE anti-human CD4 (Biolegend, 100408), APC anti-human CD8 (Biolegend, 100712), APC-Cy7 anti-human CD45 (Biolegend, 103116), APC anti-human PD-1 (Biolegend, 329908), PE/Cyanine7 anti-human Tim-3 (Biolegend, 345014), PE anti-human LAG-3 (Biolegend, 369306), and anti-ULBP2 (Abcam, ab89930). The expression of ULBP2 across the cell groups was quantified using flow cytometry.

### Immunofluorescence

Fixed fibroblasts were permeabilised with 0.1% Triton X-100 and incubated overnight at 4 °C with anti-α-SMA (Abcam, ab5831). After three washes with PBS, the samples were incubated with fluorescently labelled secondary antibodies. Nuclei were counterstained with 4,6-diamidino-2-phenylindole (DAPI) before mounting. To determine the ability of ULBP2 CAR-T cells to bind to MKN-45-mCherry cells, they were co-cultured in a 35-mm glass-bottom microwell dish for 4 h. Confocal fluorescent images were captured using a Zeiss LSM880 laser microscope.

### Histological analysis

Tissue specimens obtained from patients or mice were fixed in 10% formalin, embedded in paraffin, and cut into 5-μm sections. The sections were subsequently de-paraffinised, rehydrated, and subjected to HE, Masson’s trichrome, Sirius Red, or IHC staining. The following primary antibodies were used: anti-human ULBP2 (13133-1-AP; Proteintech) and anti-human CD8 (ab316778; Abcam). Images were acquired using the TissueFAXS Viewer. Staining intensity (0, 1, 2, and 3) and the percentage of positive cells (0–100%) were independently assessed. The histoscore (H-score) was calculated by multiplying the staining intensity with the percentage of positive cells.

### RNA extraction and real-time quantitative PCR

Total RNA was extracted from tissues or cells using TRIzol reagent and reverse transcribed into cDNA using a reverse transcription kit (Bio-Rad, 1708891). The relative expression levels of mRNAs were determined by qRT-PCR with a LightCycler instrument (Roche) using SYBR Green dye (Bio-rad, 1725124), and the results were analysed by the ΔΔCT method. The sequences of forward and reverse primers (10 μM) for human *TGF-β1* and GAPDH are listed in Table S3.

### CAF proliferation detection

CAF proliferation was assayed using a Ki-67 antibody (CST, 9449S). Following the manufacturer’s protocol, cells were harvested after 7 days of treatment, washed with PBS, and fixed with 4% formaldehyde for 15 min at room temperature. After washing with PBS, cells were permeabilised on ice with 0.1% PBS-Tween for 15 min. According to the datasheet, 1 × 10^6^ cells were incubated with the Ki-67 antibody for 1 h at room temperature, followed by washing with PBS. Before measurement, cells were again washed twice and resuspended in PBS. Measurements were performed using FCM (BD Biosciences).

### Immunoblotting

Cells (2 × 10^6^) or tissues (20 mg) were washed twice with cold PBS and whole cell lysates were extracted using 100 μL of lysis buffer (PBS, 1% NP-40, 0.5% sodium deoxycholate, 0.1% sodium dodecyl sulphate) containing phosphatase inhibitor cocktail. After lysis, the cell debris were removed by centrifugation at 12,000 × g for 10 min. The primary antibodies used for immunoblotting were anti-ULBP2 (Abcam, ab275023), anti-GAPDH (Proteintech, 10494-1-AP), anti-TGF-β1 (Selleck, F1624), anti-smad2/3 (Selleck, F0363), anti-*p*-smad2 (Selleck, A5192), anti-E-cadherin (Abcam, ab314063), anti-N-cadherin (Abcam, ab256744), and anti-Vimentin (Abcam, ab92547). Horseradish peroxidase-conjugated goat anti-mouse IgG antibody was used as a secondary antibody.

### Enzyme-linked immunosorbent assays (ELISA)

The protein levels of human IFN-γ and granzyme B in the supernatant from cytotoxicity assays of CAR-T cells against GC cells or organoids, and the protein levels of human IFN-γ, granzyme B, IL-6, IL-1β and TNF-α in the serum of CDX or PDX mice were measured using human ELISA kits (Solarbio, SEKH-0193, SEKH-0046, SEKH-0013, SEKH-0047 and SEKH-0002) following the manufacturer’s protocol. A total of 100 μL of sample or standard was added to each well and incubated on a shaker at 37 °C for 1–2 h. Then, 100 μL of biotinylated antibody and enzyme-labelled reagent were added for further incubation. After incubation, the colour-developing solution was added, and the reaction was terminated with a stop solution. The absorbance at 450 nm was measured within 10 min using a microplate reader.

### Statistical analysis

Statistical analyses were performed using SPSS 24.0 (IBM, Armonk, NY) and GraphPad Prism 8.0 (San Diego, CA). The normality of data distribution was assessed using the Shapiro–Wilk test, and the homogeneity of variances was evaluated using Levene’s test. Comparisons between two groups were performed using an unpaired two-tailed t-test or a paired t-test, as appropriate. For comparisons among multiple groups, one-way analysis of variance (ANOVA) was conducted, followed by either Tamhane’s T2 or LSD post hoc test, depending on variance equality. Survival analyses were conducted using the Kaplan–Meier method, and survival curves were compared using the log-rank (Mantel–Cox) test. Data are presented as mean ± standard error of the mean (SEM). Statistical significance was denoted as follows: *p < 0.05, **p < 0.01, and ***p < 0.001.

## Supplementary information

Supplementary_Materials

Original Western Blots

Original qPCR data

## Data Availability

All data needed to evaluate the conclusions in the paper are present in the paper and/or the Supplementary Materials. Original images of Western blots and qPCR results are provided as supplementary material. The RNA-sequencing datasets generated in this study and the code used for single-cell RNA sequencing analysis have been deposited in the Figshare repository (https://figshare.com/) and are available from the corresponding author upon reasonable request.

## References

[1] Bray F, Laversanne M, Sung H, Ferlay J, Siegel RL, Soerjomataram I, et al. Global cancer statistics 2022: GLOBOCAN estimates of incidence and mortality worldwide for 36 cancers in 185 countries. CA Cancer J Clin. 2024;74:229–63. 38572751 10.3322/caac.21834

[2] Smyth EC, Nilsson M, Grabsch HI, van Grieken NC, Lordick F. Gastric cancer. Lancet. 2020;396:635–48. 32861308 10.1016/S0140-6736(20)31288-5

[3] Arpinati L, Carradori G, Scherz-Shouval R. CAF-induced physical constraints controlling T cell state and localization in solid tumors. Nat Rev Cancer. 2024;24:676–93. 39251836 10.1038/s41568-024-00740-4

[4] Rha SY, Oh DY, Yanez P, Bai Y, Ryu MH, Lee J, et al. Pembrolizumab plus chemotherapy versus placebo plus chemotherapy for HER2-negative advanced gastric cancer (KEYNOTE-859): a multicentre, randomised, double-blind, phase 3 trial. Lancet Oncol. 2023;24:1181–95. 37875143 10.1016/S1470-2045(23)00515-6

[5] Yasuda T, Wang YA. Gastric cancer immunosuppressive microenvironment heterogeneity: implications for therapy development. Trends Cancer. 2024;10:627–42. 38600020 10.1016/j.trecan.2024.03.008 PMC11292672

[6] Maude SL, Frey N, Shaw PA, Aplenc R, Barrett DM, Bunin NJ, et al. Chimeric antigen receptor T cells for sustained remissions in leukemia. N Engl J Med. 2014;371:1507–17. 25317870 10.1056/NEJMoa1407222 PMC4267531

[7] Parikh RH, Lonial S. Chimeric antigen receptor T-cell therapy in multiple myeloma: a comprehensive review of current data and implications for clinical practice. CA Cancer J Clin. 2023;73:275–85. 36627265 10.3322/caac.21771

[8] Albelda SM. CAR T cell therapy for patients with solid tumors: key lessons to learn and unlearn. Nat Rev Clin Oncol. 2024;21:47–66. 37904019 10.1038/s41571-023-00832-4

[9] Qi C, Chong X, Zhou T, Ma M, Gong J, Zhang M, et al. Clinicopathological significance and immunotherapeutic outcome of claudin 18.2 expression in advanced gastric cancer: a retrospective study. Chin J Cancer Res. 2024;36:78–89. 38455365 10.21147/j.issn.1000-9604.2024.01.08 PMC10915633

[10] Qi C, Liu C, Gong J, Liu D, Wang X, Zhang P, et al. Claudin18.2-specific CAR T cells in gastrointestinal cancers: phase 1 trial final results. Nat Med. 2024;30:2224–34. 38830992 10.1038/s41591-024-03037-z

[11] Qin L, Wang L, Zhang J, Zhou H, Yang Z, Wang Y, et al. Therapeutic strategies targeting uPAR potentiate anti-PD-1 efficacy in diffuse-type gastric cancer. Sci Adv. 2022;8:eabn3774. 35613265 10.1126/sciadv.abn3774 PMC9132454

[12] Song Y, Tong C, Wang Y, Gao Y, Dai H, Guo Y, et al. Effective and persistent antitumor activity of HER2-directed CAR-T cells against gastric cancer cells in vitro and xenotransplanted tumors in vivo. Protein Cell. 2018;9:867–78. 28284008 10.1007/s13238-017-0384-8 PMC6160382

[13] Lv J, Zhao R, Wu D, Zheng D, Wu Z, Shi J, et al. Mesothelin is a target of chimeric antigen receptor T cells for treating gastric cancer. J Hematol Oncol. 2019;12:18. 30777106 10.1186/s13045-019-0704-y PMC6380000

[14] Han Y, Sun B, Cai H, Xuan Y. Simultaneously target of normal and stem cells-like gastric cancer cells via cisplatin and anti-CD133 CAR-T combination therapy. Cancer Immunol Immunother. 2021;70:2795–803. 33635343 10.1007/s00262-021-02891-x PMC10991976

[15] Zhao W, Jia L, Zhang M, Huang X, Qian P, Tang Q, et al. The killing effect of novel bi-specific Trop2/PD-L1 CAR-T cell targeted gastric cancer. Am J Cancer Res. 2019;9:1846–56. 31497363 PMC6726977

[16] Duan S, Guo W, Xu Z, He Y, Liang C, Mo Y, et al. Natural killer group 2D receptor and its ligands in cancer immune escape. Mol Cancer. 2019;18:29. 30813924 10.1186/s12943-019-0956-8 PMC6391774

[17] Ding H, Yang X, Wei Y. Fusion proteins of NKG2D/NKG2DL in cancer immunotherapy. Int J Mol Sci. 2018;19:177. 10.3390/ijms19010177 PMC5796126 29316666

[18] Sun B, Yang D, Dai H, Liu X, Jia R, Cui X, et al. Eradication of hepatocellular carcinoma by NKG2D-based CAR-T cells. Cancer Immunol Res. 2019;7:1813–23. 31484657 10.1158/2326-6066.CIR-19-0026

[19] Yang D, Sun B, Dai H, Li W, Shi L, Zhang P, et al. T cells expressing NKG2D chimeric antigen receptors efficiently eliminate glioblastoma and cancer stem cells. J Immunother Cancer. 2019;7:171. 31288857 10.1186/s40425-019-0642-9 PMC6617951

[20] Han Y, Xie W, Song DG, Powell DJ Jr. Control of triple-negative breast cancer using ex vivo self-enriched, costimulated NKG2D CAR T cells. J Hematol Oncol. 2018;11:92. 29980239 10.1186/s13045-018-0635-z PMC6035420

[21] Tao K, He M, Tao F, Xu G, Ye M, Zheng Y, et al. Development of NKG2D-based chimeric antigen receptor-T cells for gastric cancer treatment. Cancer Chemother Pharm. 2018;82:815–27. 10.1007/s00280-018-3670-0 30132099

[22] Zhang Y, Liang K, Zhou X, Zhang X, Xu H, Dai H, et al. Combination therapy of DKK1 inhibition and NKG2D chimeric antigen receptor T cells for the treatment of gastric cancer. Cancer Sci. 2023;114:2798–809. 37151176 10.1111/cas.15828 PMC10323088

[23] Kegasawa T, Tatsumi T, Yoshioka T, Suda T, Ikezawa K, Nakabori T, et al. Soluble UL16-binding protein 2 is associated with a poor prognosis in pancreatic cancer patients. Biochem Biophys Res Commun. 2019;517:84–8. 31303272 10.1016/j.bbrc.2019.07.020

[24] Yang X, Su X, Wang Z, Yu Y, Wu Z, Zhang D. ULBP2 is a biomarker related to prognosis and immunity in colon cancer. Mol Cell Biochem. 2023;478:2207–19. 36633827 10.1007/s11010-022-04647-2

[25] Feng R, Xu J, Huang J, Liu J, Wang X, Wang J, et al. An immune-related prognostic gene ULBP2 is correlated with immunosuppressive tumor microenvironment and immunotherapy in breast cancer. Heliyon. 2024;10:e23687. 38205308 10.1016/j.heliyon.2023.e23687 PMC10776944

[26] Peng D, Fu M, Wang M, Wei Y, Wei X. Targeting TGF-beta signal transduction for fibrosis and cancer therapy. Mol Cancer. 2022;21:104. 35461253 10.1186/s12943-022-01569-x PMC9033932

[27] Calon A, Tauriello DV, Batlle E. TGF-beta in CAF-mediated tumor growth and metastasis. Semin Cancer Biol. 2014;25:15–22. 24412104 10.1016/j.semcancer.2013.12.008

[28] Su J, Morgani SM, David CJ, Wang Q, Er EE, Huang YH, et al. TGF-beta orchestrates fibrogenic and developmental EMTs via the RAS effector RREB1. Nature. 2020;577:566–71. 31915377 10.1038/s41586-019-1897-5 PMC7450666

[29] Si J, Shi X, Sun S, Zou B, Li Y, An D, et al. Hematopoietic progenitor kinase1 (HPK1) mediates T cell dysfunction and is a druggable target for T cell-based immunotherapies. Cancer Cell. 2020;38:551–66.e11. 32860752 10.1016/j.ccell.2020.08.001

[30] Hidalgo M, Amant F, Biankin AV, Budinska E, Byrne AT, Caldas C, et al. Patient-derived xenograft models: an emerging platform for translational cancer research. Cancer Discov. 2014;4:998–1013. 25185190 10.1158/2159-8290.CD-14-0001 PMC4167608

[31] Michaux A, Mauen S, Breman E, Dheur MS, Twyffels L, Saerens L, et al. Clinical grade manufacture of CYAD-101, a NKG2D-based, first in class, non-gene-edited allogeneic CAR T-cell therapy. J Immunother. 2022;45:150–61. 35191428 10.1097/CJI.0000000000000413

[32] Sallman DA, Kerre T, Havelange V, Poire X, Lewalle P, Wang ES, et al. CYAD-01, an autologous NKG2D-based CAR T-cell therapy, in relapsed or refractory acute myeloid leukaemia and myelodysplastic syndromes or multiple myeloma (THINK): haematological cohorts of the dose escalation segment of a phase 1 trial. Lancet Haematol. 2023;10:e191–e202. 36764323 10.1016/S2352-3026(22)00378-7

[33] Chong X, Madeti Y, Cai J, Li W, Cong L, Lu J, et al. Recent developments in immunotherapy for gastrointestinal tract cancers. J Hematol Oncol. 2024;17:65. 39123202 10.1186/s13045-024-01578-x PMC11316403

[34] de Kruijf EM, Sajet A, van Nes JG, Putter H, Smit VT, Eagle RA, et al. NKG2D ligand tumor expression and association with clinical outcome in early breast cancer patients: an observational study. BMC Cancer. 2012;12:24. 22257486 10.1186/1471-2407-12-24 PMC3292504

[35] McGilvray RW, Eagle RA, Rolland P, Jafferji I, Trowsdale J, Durrant LG. ULBP2 and RAET1E NKG2D ligands are independent predictors of poor prognosis in ovarian cancer patients. Int J Cancer. 2010;127:1412–20. 20054857 10.1002/ijc.25156

[36] Li K, Mandai M, Hamanishi J, Matsumura N, Suzuki A, Yagi H, et al. Clinical significance of the NKG2D ligands, MICA/B and ULBP2 in ovarian cancer: high expression of ULBP2 is an indicator of poor prognosis. Cancer Immunol Immunother. 2009;58:641–52. 18791713 10.1007/s00262-008-0585-3 PMC11030581

[37] Fernandez-Messina L, Ashiru O, Aguera-Gonzalez S, Reyburn HT, Vales-Gomez M. The human NKG2D ligand ULBP2 can be expressed at the cell surface with or without a GPI anchor and both forms can activate NK cells. J Cell Sci. 2011;124:321–7. 21224393 10.1242/jcs.076042 PMC3021996

[38] Wang R, Sun PD. Natural killer cell-mediated shedding of ULBP2. PLoS One. 2014;9:e91133. 24614922 10.1371/journal.pone.0091133 PMC3948742

[39] Peng YP, Zhu Y, Zhang JJ, Xu ZK, Qian ZY, Dai CC, et al. Comprehensive analysis of the percentage of surface receptors and cytotoxic granules positive natural killer cells in patients with pancreatic cancer, gastric cancer, and colorectal cancer. J Transl Med. 2013;11:262. 24138752 10.1186/1479-5876-11-262 PMC3854023

[40] Song H, Kim J, Cosman D, Choi I. Soluble ULBP suppresses natural killer cell activity via down-regulating NKG2D expression. Cell Immunol. 2006;239:22–30. 16630603 10.1016/j.cellimm.2006.03.002

[41] Groh V, Wu J, Yee C, Spies T. Tumor-derived soluble MIC ligands impair expression of NKG2D and T-cell activation. Nature. 2002;419:734–8. 12384702 10.1038/nature01112

[42] Lee YS, Choi H, Cho HR, Son WC, Park YS, Kang CD, et al. Downregulation of NKG2DLs by TGF-beta in human lung cancer cells. BMC Immunol. 2021;22:44. 34253166 10.1186/s12865-021-00434-8 PMC8273967

[43] Lee JC, Lee KM, Ahn YO, Suh B, Heo DS. A possible mechanism of impaired NK cytotoxicity in cancer patients: down-regulation of DAP10 by TGF-beta1. Tumori. 2011;97:350–7. 21789015 10.1177/030089161109700316

[44] Obajdin J, Larcombe-Young D, Glover M, Kausar F, Hull CM, Flaherty KR, et al. Solid tumor immunotherapy using NKG2D-based adaptor CAR T cells. Cell Rep Med. 2024;5:101827. 39566469 10.1016/j.xcrm.2024.101827 PMC11604534

[45] Parihar R, Rivas C, Huynh M, Omer B, Lapteva N, Metelitsa LS, et al. NK cells expressing a chimeric activating receptor eliminate MDSCs and rescue impaired CAR-T cell activity against solid tumors. Cancer Immunol Res. 2019;7:363–75. 30651290 10.1158/2326-6066.CIR-18-0572 PMC7906796

[46] Baumeister SH, Murad J, Werner L, Daley H, Trebeden-Negre H, Gicobi JK, et al. Phase I trial of autologous CAR T cells targeting NKG2D ligands in patients with AML/MDS and multiple myeloma. Cancer Immunol Res. 2019;7:100–12. 30396908 10.1158/2326-6066.CIR-18-0307 PMC7814996

[47] Yang D, Sun B, Li S, Wei W, Liu X, Cui X, et al. NKG2D-CAR T cells eliminate senescent cells in aged mice and nonhuman primates. Sci Transl Med. 2023;15:eadd1951. 37585504 10.1126/scitranslmed.add1951

[48] Philp LK. Patient-derived xenograft models for translational prostate cancer research and drug development. Methods Mol Biol. 2024;2806:153–85. 38676802 10.1007/978-1-0716-3858-3_12

[49] Lee SH, Hu W, Matulay JT, Silva MV, Owczarek TB, Kim K, et al. Tumor evolution and drug response in patient-derived organoid models of bladder cancer. Cell. 2018;173:515–28.e17. 29625057 10.1016/j.cell.2018.03.017 PMC5890941

