# Supplementary material for: ULBP2 CAR-T cells enhance gastric cancer immunotherapy by inhibiting CAF activation
Source: Cell Death Dis. 2025 Aug 8;16(1):597. doi: 10.1038/s41419-025-07905-5 (PMC12332075; doi:10.1038/s41419-025-07905-5)
Supplement: Supplementary file 1 — Supplementary_Materials [file 41419_2025_7905_MOESM1_ESM.docx]

**Supplementary Figure Legends**


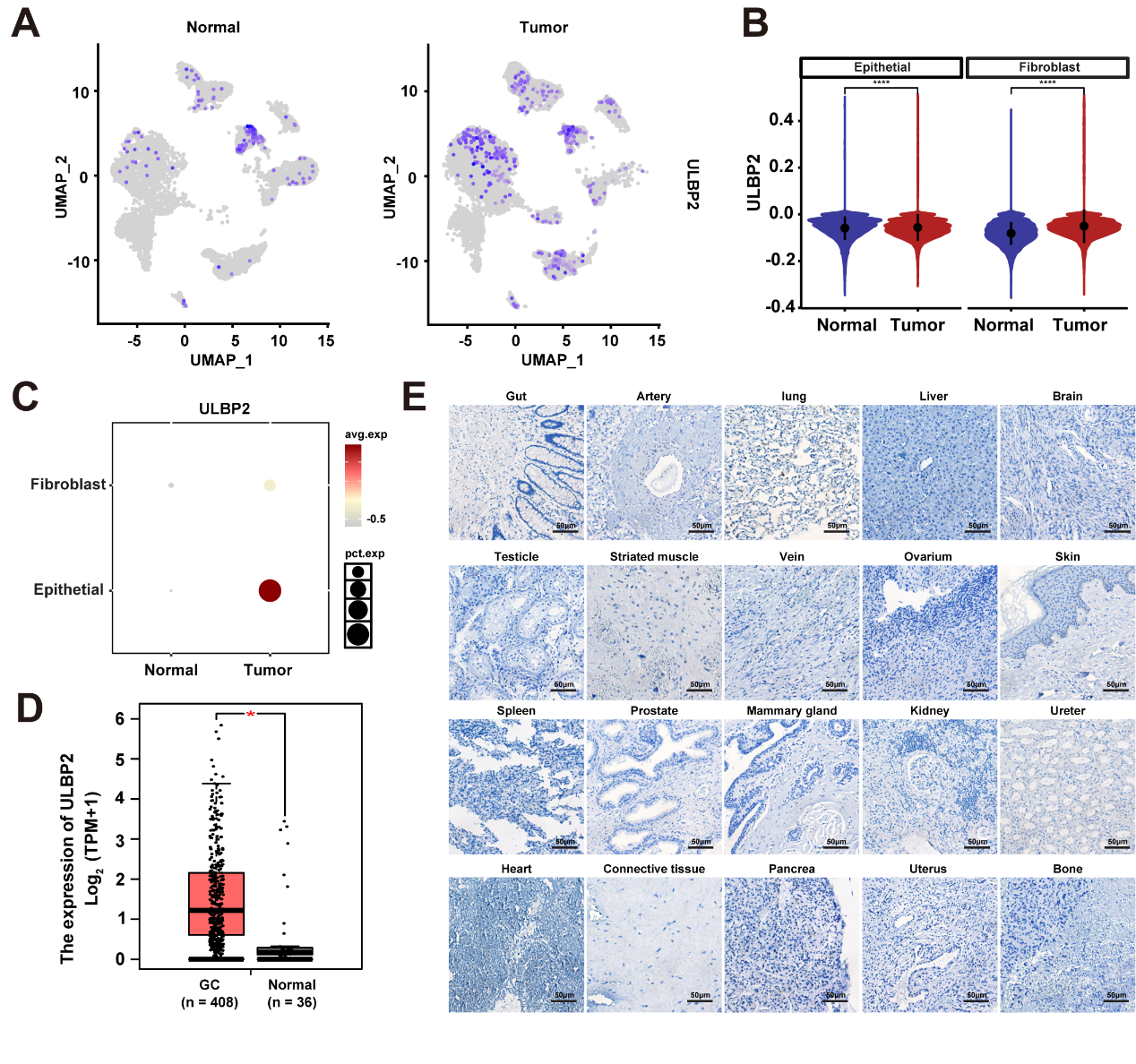
 **Figure S1. Related to Figure 1.** (A) t-SNE plots showing the ULBP2 expression in GC tumour and normal tissues. (B and C) ULBP2 expression in epithelial cells and fibroblasts from tumour and normal tissues analysed in the GSE163558 and GSE206785 datasets. (D) ULBP2 expression in TCGA GC database. (E) IHC analysis of ULBP2 protein levels in human vital organs. Data are expressed as the mean ± SEM (*p < 0.05, ***p < 0.001).

**
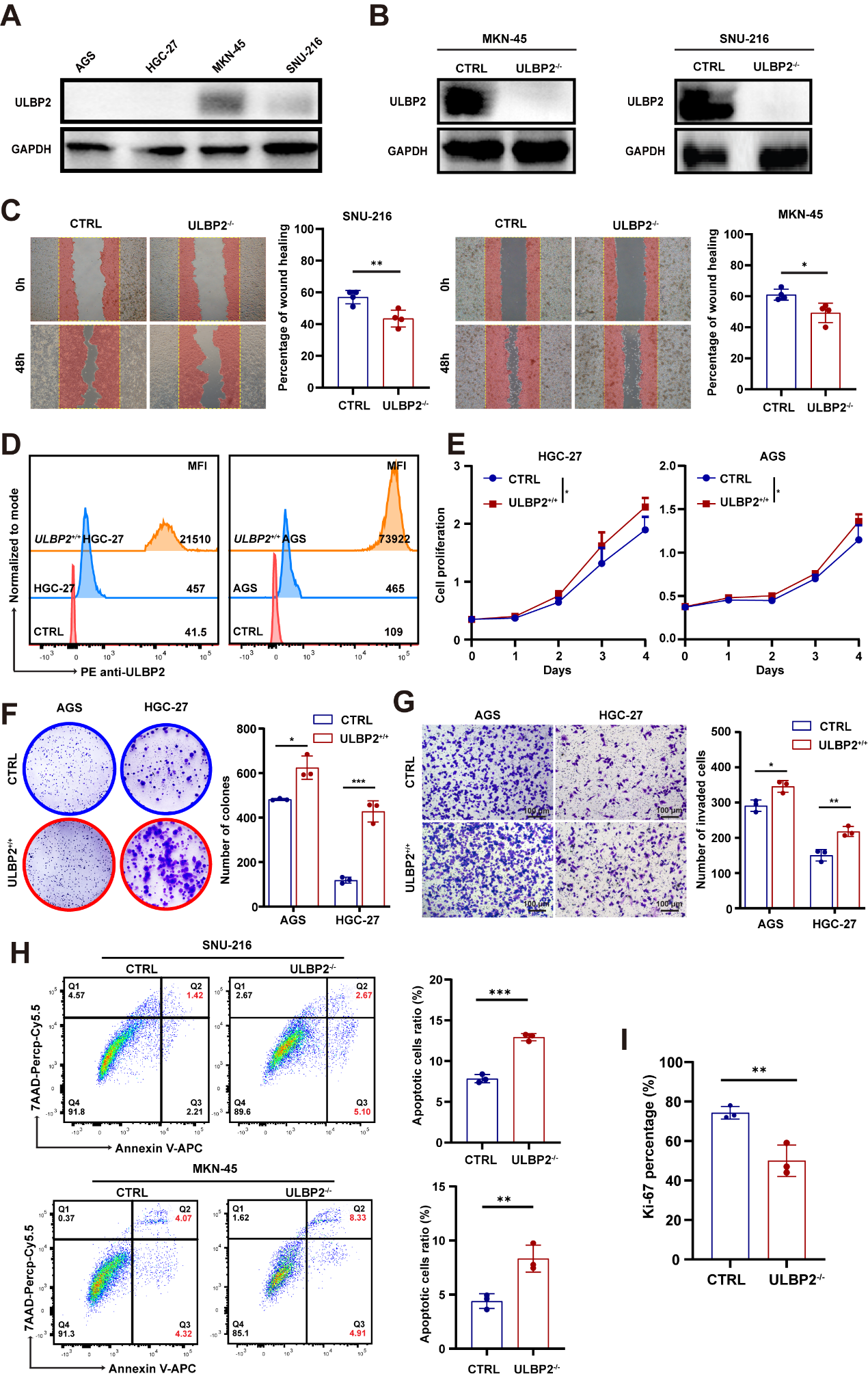
 Figure S2. Related to Figure 2.** (A) ULBP2 protein levels in GC cell lines shown by western blotting. (B) ULBP2 knockout efficiency in MKN-45 and SNU-216 shown by western blotting. (C) Wound healing assay measuring the healing rate of GC cells with corresponding *ULBP2^-/-^* cells (n = 4). (D) *ULBP2* overexpression efficiency in HGC-27 and AGS cells shown by FCM. (E) Growth curves of ctrl cells and *ULBP2*^+/+^ HGC-27 and AGS cells (n = 3). (F) Colony formation assay and the statistical results of ctrl cells and *ULBP2*^+/+^ HGC-27 and AGS cells (n = 3). (G) Transwell invasion assay of ctrl cells and *ULBP2*^+/+^ HGC-27 and AGS cells (n = 3). (H) FCM revealed that *ULBP2* knockout could significantly induce the apoptosis of SNU-216 and MKN-45 cells (n = 3). (I) Quantification of Ki67 in NSG mice carrying wild type, *ULBP2*^-/-^ MKN-45 CDX (n = 3). Data are expressed as the mean ± SEM (*p < 0.05, **p < 0.01, ***p < 0.001).

**
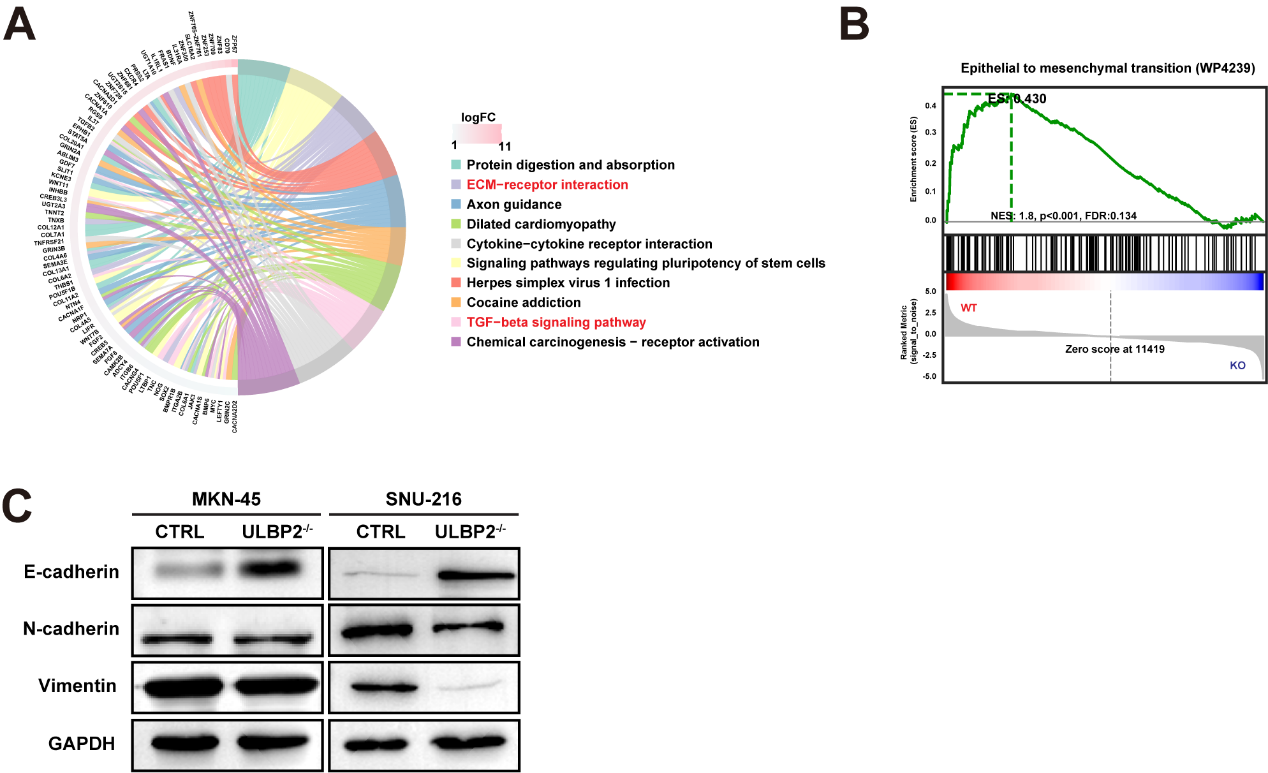
 Figure S3. Related to Figure 3.** (A) Gene Ontology (GO) analysis of transcriptomic differences between *ULBP2^-/-^* MKN-45 cells and wild type cells. (B) Transcriptome GSEA analysis revealed that the differentially expressed genes were significantly enriched in Epithelial to mesenchymal transition (EMT). (C) Western blotting of E-cadherin, N-cadherin, and Vimentin in ctrl cells and *ULBP2*^-/-^ MKN-45 and SNU-216 cells.


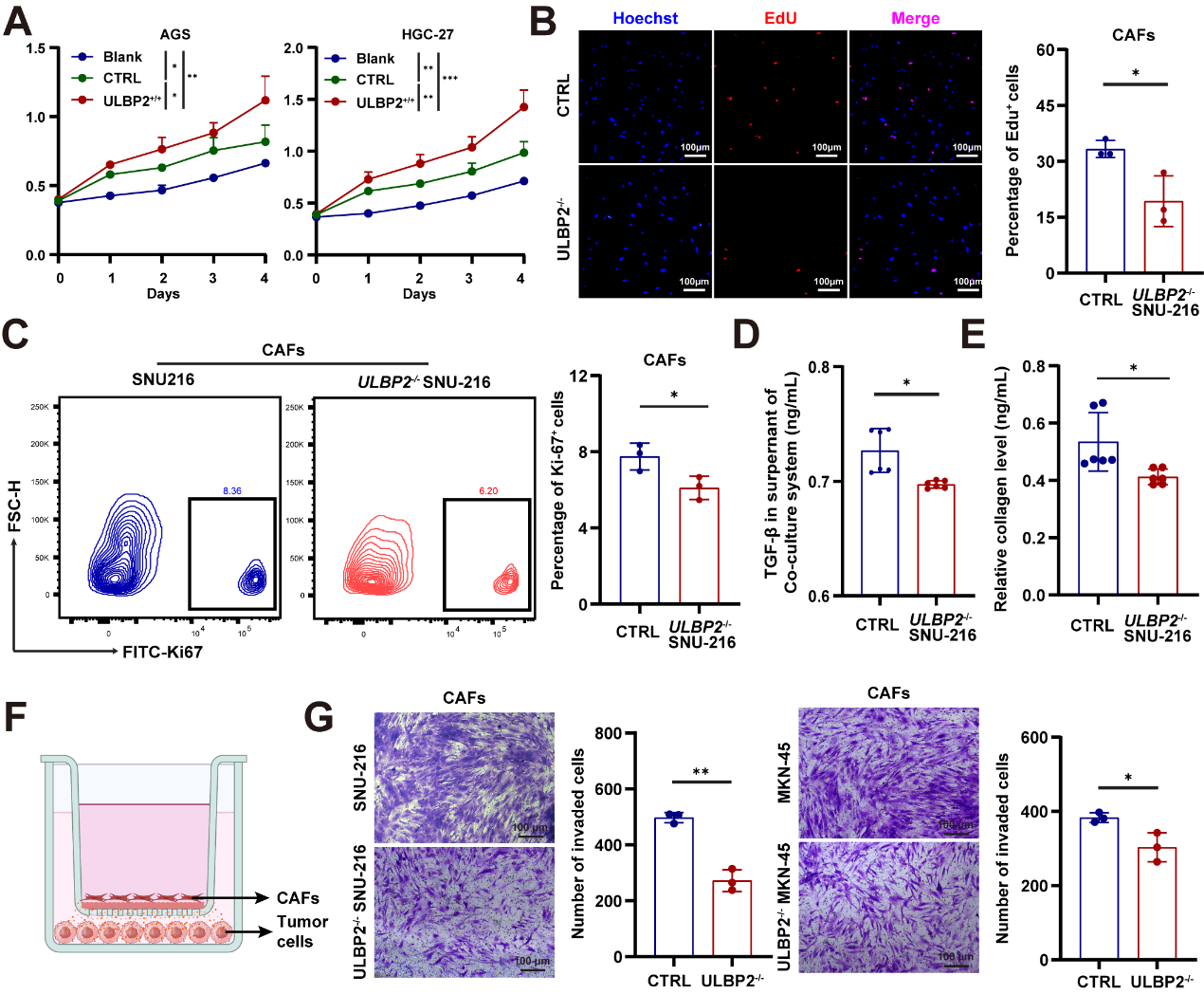
 **Figure S4. Related to Figure 4.** (A) Growth curves of CAFs cultured with supernatants from ctrl cells and *ULBP2*^+/+^ AGS and HGC-27 cells (n = 3). (B) EdU assay showed that the CAFs proliferation was significantly reduced in the co-culture system of *ULBP2* knockout SNU-216 cells compared with wild type cells (n = 3). (C) Frequency of Ki67 in the co-culture system, as shown by FCM (n = 3). (D and E) Relative TGF-β1 (D) (n = 6) and collagen (E) (n = 6) content in the co-culture system. (F) The diagram of co-culture system between CAFs and GC cells. (G) Transwell invasion assay of CAFs in co-culture system (n = 3). Data are expressed as mean ± SEM (*p < 0.05, **p < 0.01, ***p < 0.001).

**
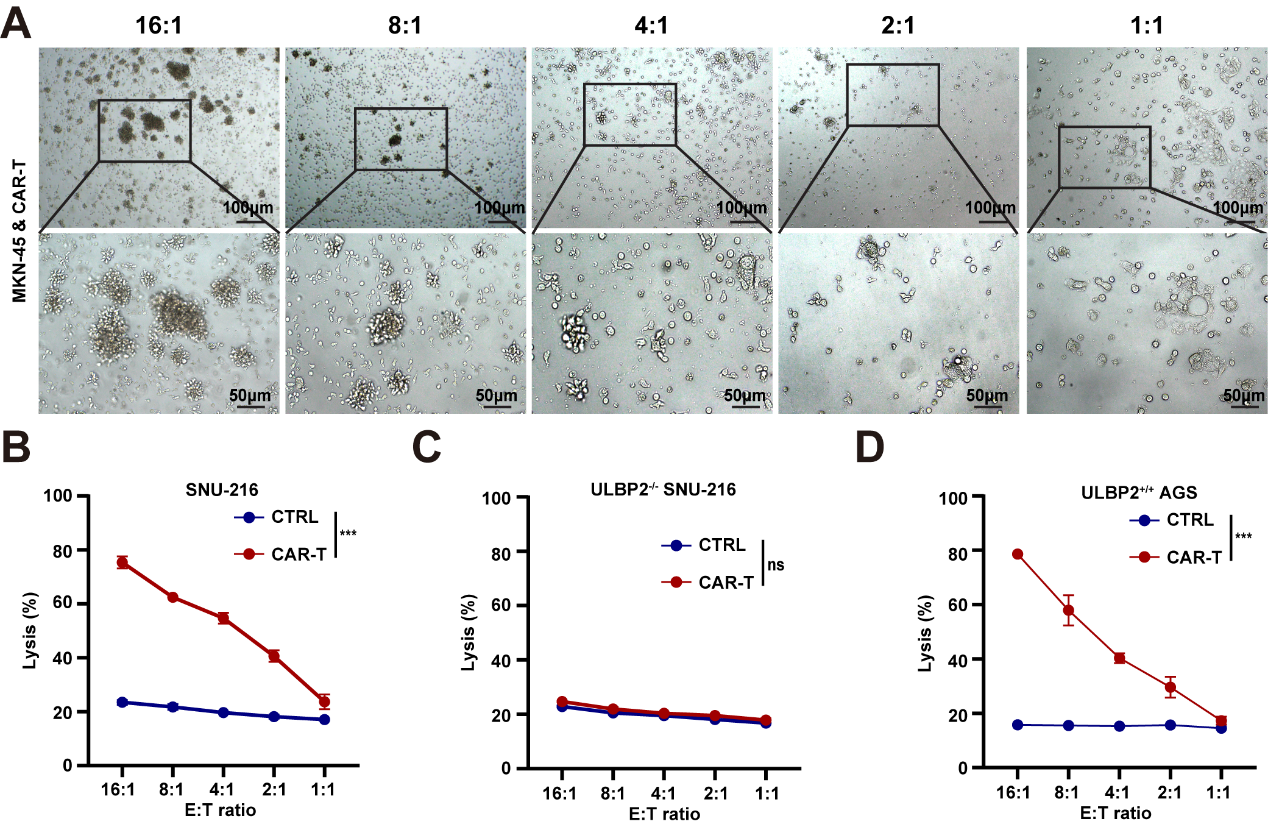
 Figure S5. Related to Figure 5.** (A) Representative images of MKN-45 cells co-cultured with ULBP2 CAR-T cells. E:T, effector cells: target cells. (B to D) Killing efficiency of SNU-216 cells (B) (n = 3), *ULBP2*^-/-^ SNU-216 cells (C) (n = 3), and *ULBP2*^+/+^ AGS cells (D) (n = 3) by ULBP2 CAR-T cells. Data are represented as mean ± SEM (ns, nonsignificant; ***p < 0.001).

**
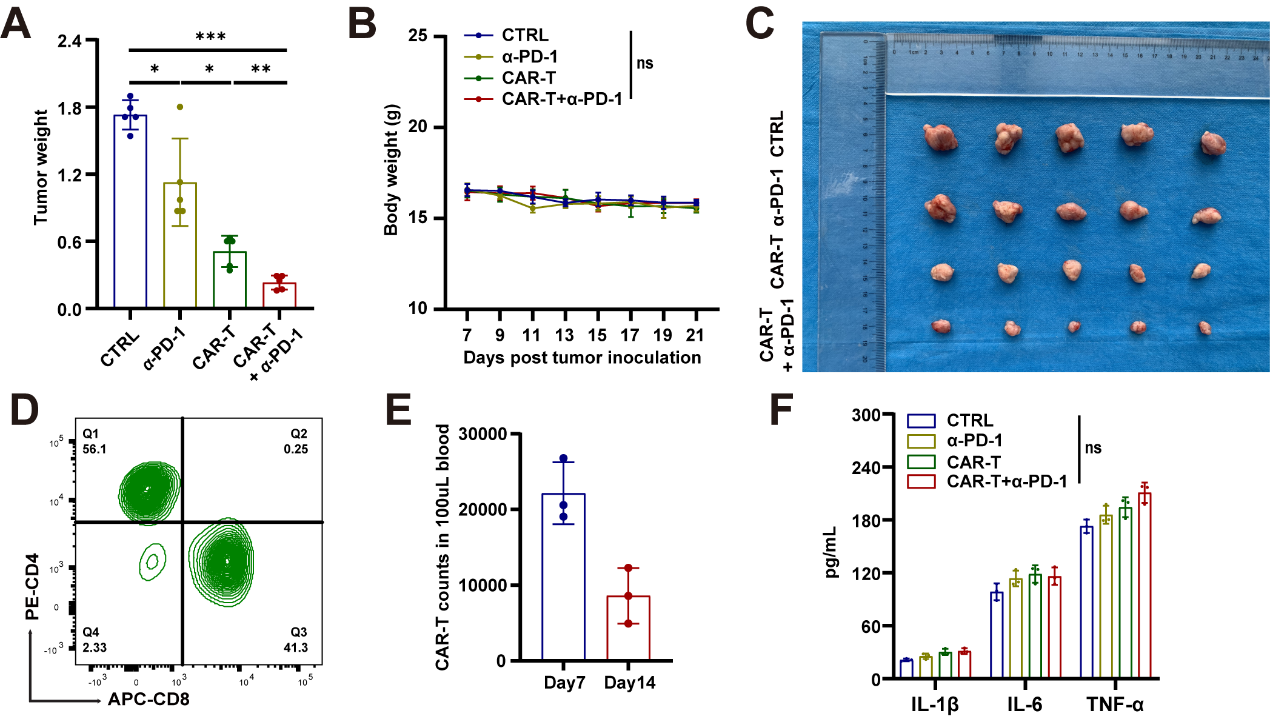
 Figure S6. Related to Figure 6.** (A) Tumour weight at day 21 post tumour inoculation (n = 5). (B) Body weight of mice bearing the CDX over the course of the experiment (n = 5). (C) Representative images of tumours at day 21 post tumour inoculation. (D) Frequencies of CD8^+^T cells and CD4^+^T cells at day 21 post tumor inoculation. (E) ULBP2 CAR-T cell counts in peripheral blood on days 7 and 14 post-infusion (n = 3). (F) Protein levels of IL-1β, IL-6 and TNF-α in the serum of CDX mice at different weeks post tumor inoculation detected by ELISA (n = 3). Data are represented as mean ± SEM (ns, nonsignificant; *p < 0.05, **p < 0.01, ***p < 0.001).


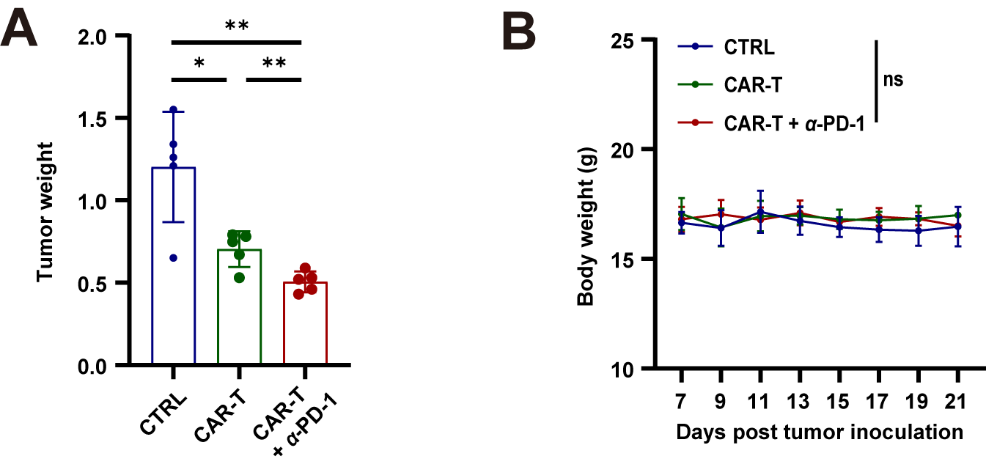


**Figure S7. Related to Figure 7.** (A) Tumour weight at day 21 post tumour inoculation (n = 5). (B) Body weight of mice bearing the PDX over the course of the experiment (n = 5). Data are represented as mean ± SEM (ns, nonsignificant; **p < 0.01, ***p < 0.001).

**Table S1. Clinical characteristics of gastric patients.**

| Clinical characterisics | GC patient |
| --- | --- |
| Age (year) | 60 |
| Gender | Male |
| Serum protein | TP (49.6 g/L) |
|  | ALB (39.9 g/L) |
|  | CA199 (12.70 U/ml) |
|  | CA125 (12.40 U/ml) |
|  | CEA (2.39 ng/ml) |
|  | AFP (4.11 ng/ml) |
| Neoadjuvant chemotherapy regimen | None |
| Operation date | 2023.11.29 |
| Operation style | Laparoscopic Gastric Lesion Resection |
| Operation time (min) | 180 min |
| Tumour location | Upper third |
| Tumour size | 5×4.5×1.2 cm |
| Lauren classification | mixed type |
| TNM stage | T3N0M0 |
| Immunohistochemistry | CK8/18 (+) |
|  | P53 (-) |
|  | C-erbB-2 (+) |
|  | PMS-2 (+) |
|  | MLH-1 (+) |
|  | MSH-6 (+) |
|  | MSH-2 (+) |
|  | Syn (-) |
| Ki67 | 60% |
| Postoperative chemotherapy regimen | Two cycles of SOX |
| Postoperative survival | Alive |

**Table S2. Sequences of the guide RNA (gRNA) and overexpression (OE).**

| Target | Sequences (5’-3’) |
| --- | --- |
| gRNA | CTGTCCGGCTGGTCCCGGGC(TGG) |
| OE-Fw | TACAAAGACGATGACGATAAGATGGCAGCAGCCGCCGCTACC |
| OE-Rv | TAGAAGACTTCCTCTGCCCTCGATGCCAGGGAGGATGAAGCA |

**Table S3. Primer sequences used for qRT-PCR.**

| Gene | Sequences (5’-3’) |
| --- | --- |
| GAPDH-Forward | GGAGCGAGATCCCTCCAAAAT |
| GAPDH-Reverse | GGCTGTTGTCATACTTCTCATGG |
| TGF-β1-Forward | CTAATGGTGGAAACCCACAACG |
| TGF-β1-Reverse | TATCGCCAGGAATTGTTGCTG |

**Table S4: Antibodies used in the study.**

| Antibody | Manufacturer | Cat. No. | Application | Dilution |
| --- | --- | --- | --- | --- |
| ULBP2 | Abcam | ab275023 | WB | 1:1000 |
| ULBP2 | Proteintech | 13133-1-AP | IHC | 1:1000 |
| ULBP2 | Abcam | ab89930 | FC | 1 µg/ml |
| PE Goat anti-mouse IgG | BioLegend | 405307 | FC | 0.2 mg/ml |
| TGFβ1 | Selleck | F1624 | WB | 1:1000 |
| T-Smad2/3 | Selleck | F0363 | WB | 1:1000 |
| p-Smad2 | Selleck | A5192 | WB | 1:1000 |
| GAPDH | Proteintech | 10494-1-AP | WB | 1:5000 |
| α-SMA | Abcam | ab5831 | IF | 1:100 |
| Ki-67 | CST | 9449S | IHC/IF | 1:1600 |
| Ki-67 | CST | 9449S | ICFC | 1:100 |
| CD8 | Abcam | ab316778 | IHC/IF | 1:100 |
| E-Cad | Abcam | ab314063 | WB | 1:1000 |
| N-Cad | Abcam | ab256744 | WB | 1:1000 |
| Vimentin | Abcam | ab92547 | WB | 1:1000 |
| APC-Cy7 anti-human CD45 | BioLegend | 103116 | FC | 5 µl/100 µl |
| FITC anti-human CD3 | BioLegend | 100204 | FC | 5 µl/100 µl |
| PE anti-human CD4 | BioLegend | 100408 | FC | 5 µl/100 µl |
| APC anti-human CD8 | BioLegend | 100712 | FC | 5 µl/100 µl |
| APC anti-human PD-1 | BioLegend | 329908 | FC | 5 µl/100 µl |
| PE/Cyanine7 anti-human Tim3 | BioLegend | 345014 | FC | 5 µl/100 µl |
| PE anti-human LAG-3 | BioLegend | 369306 | FC | 5 µl/100 µl |
| Goat Anti-Rabbit IgG Alexa488 | Abcam | ab150077 | IF | 1:200 |
| Goat Anti-Mouse IgG Alexa488 | Abcam | ab150113 | IF | 1:200 |
| Goat Anti-Mouse IgG Alexa594 | Abcam | ab150116 | IF | 1:200 |
