# Supplementary material for: ULBP2 CAR-T cells enhance gastric cancer immunotherapy by inhibiting CAF activation
Source: Cell Death Dis. 2025 Aug 8;16(1):597. doi: 10.1038/s41419-025-07905-5 (PMC12332075; doi:10.1038/s41419-025-07905-5)
Supplement: Supplementary file 2 — Original Western Blots [file 41419_2025_7905_MOESM2_ESM.docx]

**Figure1**

The Protein level of ULBP2 across the Gastric Samples(GC&AN)

Case#1ULBP2 Case#1GAPDH





Case#2ULBP2 Case#2GAPDH







Case#3ULBP2 Case#3GAPDH







Case#4ULBP2 Case#4GAPDH







Case#5ULBP2 Case#5GAPDH







Case#6ULBP2 Case#6GAPDH







**Figure3**

The protein within TGF-β pathway of MKN45&SNU216(WT VS KO)

MKN45-WT&KO_TGFβ1 MKN45-WT&KO_t-SMAD2/3







MKN45-WT&KO_*p*-SMAD2 MKN45-WT&KO_GAPDH







**Figure3**

The protein within TGF-β pathway of MKN45&SNU216(WT VS KO)

SNU216-WT&KO_TGFβ1 SNU216-WT&KO_t-SMAD2/3




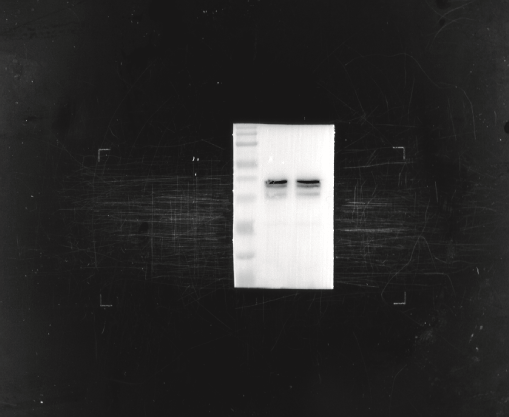


SNU216-WT&KO_*p*-SMAD2 SNU216-WT&KO_GAPDH







**Fig. S2.**

The Protein level of ULBP2 across the Gastric tumor cell lines

ULBP2





GAPDH





Monoclonal validation of MKN45 ULBP2^-/-^

ULBP2 GAPDH

^

^ **

**

Monoclonal validation of SNU216 ULBP2^-/-^

ULBP2 GAPDH







**Fig. S3.**

**EMT of MKN45**

E-Cad N-Cad




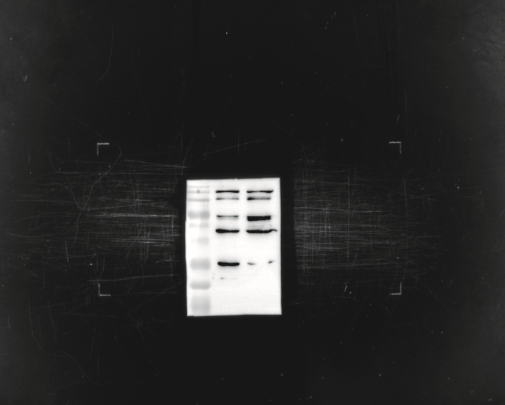


Vimentin GAPDH


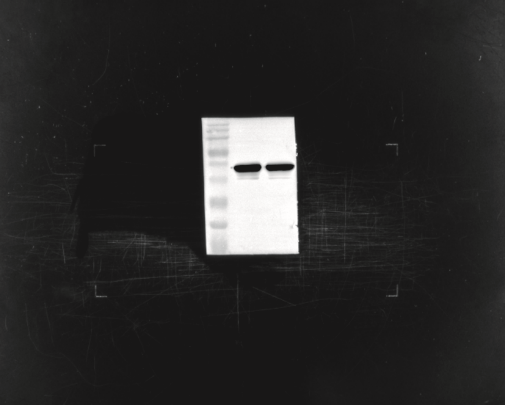




**EMT of SNU216**

E-Cad N-Cad







Vimentin GAPDH
