# Supplementary material for: ULBP2 CAR-T cells enhance gastric cancer immunotherapy by inhibiting CAF activation
Source: Cell Death Dis. 2025 Aug 8;16(1):597. doi: 10.1038/s41419-025-07905-5 (PMC12332075; doi:10.1038/s41419-025-07905-5)
Supplement: Supplementary file 3 — Original qPCR data [file 41419_2025_7905_MOESM3_ESM.docx]

**Figure3**

The mRNA level of TGF-β1 in ctrl cells and ULBP2^-/-^ MKN-45 and SNU-216 cells

| Target | dye | Sample | CT |
| --- | --- | --- | --- |
| TGFβ1 | SYBR | KOMKN45 | 25.60 |
| TGFβ1 | SYBR | KOMKN45 | 25.69 |
| TGFβ1 | SYBR | KOMKN45 | 25.47 |
| TGFβ1 | SYBR | WTMKN45 | 21.14 |
| TGFβ1 | SYBR | WTMKN45 | 21.23 |
| TGFβ1 | SYBR | WTMKN45 | 21.19 |
| TGFβ1 | SYBR | KOMKN45 | 26.15 |
| TGFβ1 | SYBR | KOMKN45 | 26.00 |
| TGFβ1 | SYBR | KOMKN45 | 26.80 |
| TGFβ1 | SYBR | WTMKN45 | 23.29 |
| TGFβ1 | SYBR | WTMKN45 | 24.11 |
| TGFβ1 | SYBR | WTMKN45 | 23.93 |
| GAPDH | SYBR | KOSNU216 | 18.19 |
| GAPDH | SYBR | KOSNU216 | 18.11 |
| GAPDH | SYBR | KOSNU216 | 18.27 |
| GAPDH | SYBR | WTSNU216 | 17.32 |
| GAPDH | SYBR | WTSNU216 | 16.26 |
| GAPDH | SYBR | WTSNU216 | 15.20 |
| GAPDH | SYBR | KOSNU216 | 20.57 |
| GAPDH | SYBR | KOSNU216 | 19.39 |
| GAPDH | SYBR | KOSNU216 | 20.35 |
| GAPDH | SYBR | WTSNU216 | 19.35 |
| GAPDH | SYBR | WTSNU216 | 19.56 |
| GAPDH | SYBR | WTSNU216 | 19.55 |
